# Transcriptional profiling of liver in riboflavin-deficient chicken embryos explains impaired lipid utilization, energy depletion, massive hemorrhaging, and delayed feathering

**DOI:** 10.1186/s12864-018-4568-2

**Published:** 2018-03-05

**Authors:** Larry A. Cogburn, Danielle N. Smarsh, Xiaofei Wang, Nares Trakooljul, Wilfrid Carré, Harold B. White

**Affiliations:** 1 Department of Animal and Food Sciences, University of Delaware, Newark, DE 19716 USA; 2 Department of Chemistry and Biochemistry, University of Delaware, Newark, DE 19716 USA; 3 Present Address: Department of Animal Science, The Pennsylvania State University, University Park, PA 16802 USA; 4 Present Address: Department of Biological Sciences, Tennessee State University, Nashville, TN 37209 USA; 5 Present Address: Leibniz Institute for Farm Animal Biology (FBN), Institute for Genome Biology, Wilhelm-Stahl-Allee 2, 18196 Dummerstorf, Germany; 6 Present Address: Laboratoire de Génétique Moléculaire et Génomique, CHU Pontchaillou, 35033 Rennes, France

**Keywords:** Microarray analysis, Riboflavin deficiency, Expression profiling, Impaired lipid metabolism, β-oxidation, Protein catabolism, Protease inhibitors, Coagulation factors, Acetyl CoA deficiency, Sudden embryonic death, Chicken flavoproteome, Feather keratin

## Abstract

**Background:**

A strain of Leghorn chickens (*rd/rd*), unable to produce a functional riboflavin-binding protein, lays riboflavin-deficient eggs, in which all embryos suddenly die at mid-incubation (days 13-15). This malady, caused by riboflavin deficiency, leads to excessive lipid accumulation in liver, impaired β-oxidation of lipid, and severe hypoglycemia prior to death. We have used high-density chicken microarrays for time-course transcriptional scans of liver in chicken embryos between days 9-15 during this riboflavin-deficiency-induced metabolic catastrophe. For comparison, half of *rd/rd* embryos (*n* = 16) were rescued from this calamity by injection of riboflavin just prior to incubation of fertile eggs from *rd/rd* hens.

**Results:**

No significant differences were found between hepatic transcriptomes of riboflavin-deficient and riboflavin-rescued embryos at the first two ages (days 9 and 11). Overall, we found a 3.2-fold increase in the number of differentially expressed hepatic genes between day 13 (231 genes) and day 15 (734 genes). Higher expression of genes encoding the chicken flavoproteome was more evident in rescued- (15 genes) than in deficient-embryos (4 genes) at day 15. Diminished activity of flavin-dependent enzymes in riboflavin-deficient embryos blocks catabolism of yolk lipids, which normally serves as the predominant source of energy required for embryonic development.

**Conclusions:**

Riboflavin deficiency in mid-stage embryos leads to reduced expression of numerous genes controlling critical functions, including β-oxidation of lipids, blood coagulation and feathering. Surprisingly, reduced expression of *feather keratin 1* was found in liver of riboflavin-deficient embryos at e15, which could be related to their delayed feathering and sparse clubbed down. A large number of genes are expressed at higher levels in liver of riboflavin-deficient embryos; these up-regulated genes control lipid storage/transport, gluconeogenesis, ketogenesis, protein catabolism/ubiquitination and cell death*.*

**Electronic supplementary material:**

The online version of this article (10.1186/s12864-018-4568-2) contains supplementary material, which is available to authorized users.

## Background

The 21-day development of a chicken embryo represents the genetically and molecularly choreographed transformation of a fertilized egg into a complex living organism [1]. The cleidoic egg of birds is a self-contained, life-support system packaged by the hen that requires only external heat and oxygen for development of the embryo that hatches into a fully-functional, free-living, hatchling. Within the protective shell and surrounding the yolk, the egg white (albumen) provides a protein-rich antimicrobial barrier [2]. From our global transcriptional analysis of the hen’s oviduct during formation of the egg, we have described the orchestration of gene expression during fabrication of the eggshell membranes and calcification of the egg shell during the egg’s transit through each segment of the hen’s oviduct [3, 4]. Upon incubation, the fertilized ovum develops into an embryo at the surface of the nutrient-rich yolk from which it derives energy almost exclusively from the oxidation of lipids [5, 6]. The deficiency of a single trace nutrient, such as riboflavin, creates a catastrophic metabolic crisis that abruptly aborts the embryo’s developmental process.

**Fig. 1:**
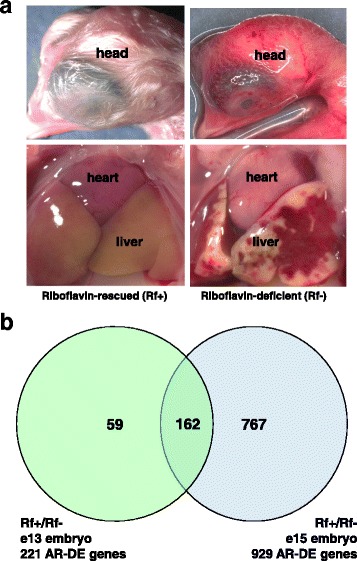
**A** Gross anatomy of riboflavin-deficient (A) and riboflavin-rescued (B) chick embryos on embryonic day 15 (e15). The riboflavin-deficient (Rf-) embryos present with extensive hemorrhage of skin, liver and heart, retarded development of the eye, feathers, beak and cranium, and excessive lipid deposition in the liver. On the other hand, the riboflavin-rescued (Rf+) embryos show normal development of the head, feathers and liver, which typically shows yellowing due to storage and utilization of yolk lipids. Gross anatomy of Rf + and Rf- embryos at e15 was documented using a Leica M420 macroscope (Buffalo Grove, IL) fitted with a cold light source and the Spot Insight 4 imaging system. The photomicrographs were taken at a magnification of 10X. **B** Venn diagram of differentially-expressed (DE) and “Analysis Ready” (AR) genes mapped to the Ingenuity Knowledge Base using Ingenuity® Pathway Analysis (IPA) software. Statistical analysis showed no DE (FDR ≤ 0.05) genes between the Rf + and Rf- embryos on e9 and e11. Of the 395 DE genes identified in the liver at e13, only 221 DE genes (or 56%) were considered by IPA as “Analysis Ready” (AR), which reflects incomplete annotation of the Roslin chicken oligo printed on this array. Likewise, 1467 DE genes were identified from statistical analysis on e15; and of these genes, only 929 (or 63%) were mapped to the Ingenuity® Knowledge Base (i.e., AR-DE genes). While only 162 AR-DE genes were commonly shared between riboflavin treatment groups across e13 and e15

Hens from a strain of White Leghorn chickens are unable to deposit riboflavin in their eggs due to a recessive mutation (*rd*) [7, 8]. A single base substitution in the donor splice site of intron 3 of the gene encoding the riboflavin-binding protein (RBP) [9] causes skipping of exon 3 [10], which subsequently prevents synthesis of a functional RBP in the hen’s liver and oviduct for deposition of riboflavin into the yolk and albumen layers [11]. Small amounts of riboflavin, in its flavin-adenine dinucleotide (FAD) coenzyme form and associated with the enzyme quiescin sulfhydryl oxidase (QSOX) [12], are present in these eggs [13], this sustains riboflavin-deficient embryos until death on day 13-15 of embryonation (e13-e15). Affected embryos have delayed and aberrant feather development (clubbed down), fatty livers and extensive cutaneous hemorrhaging (Fig. 1a). Riboflavin-deficient (Rf-) fertilized eggs are rescued by injecting a small amount of more-soluble flavin coenzyme, riboflavin-5′-phosphate (FMN), into the albumen prior to onset of incubation [7]. After hatching, the riboflavin-rescued (Rf+) chicks absorb adequate amounts of riboflavin from their diets and develop normally to maturity. We found higher (normal) expression of numerous flavin-dependent genes in liver of Rf + embryos at e15. Thus, this lethal mutation prevents riboflavin transport from liver to the oviduct for delivery into the egg. However, a single injection of FMN into the fertilized egg from *rd/rd* hens prior to onset of incubation (e0) ameliorates this metabolic crisis. Redox coenzymes derived from riboflavin (FMN and FAD) are essential components of numerous flavoprotein enzymes that reduce molecular oxygen directly, as with QSOX, or indirectly via the mitochondrial electron transport system that generates ATP. Riboflavin-deficient embryos experience a metabolic crisis when trace amounts of riboflavin become depleted and are insufficient to meet the metabolic demands on these enzymes [14]. Although Rf- embryos seem to grow normally with only a slight reduction in body weight on the day before death, signs of metabolic disruption appear around e10 when normal chick embryos become almost totally dependent on catabolism of triglycerides stored in the yolk for metabolic energy [15]. The five-fold increase in the activity of an essential flavin-dependent enzyme in the beta oxidation of fatty acids, medium-chain acyl-Coenzyme A dehydrogenase (MCAD), in liver and heart of normal embryos reflects this major metabolic switch [16]. However, with the limited riboflavin stores depleted, MCAD activity remains unchanged between day 10 and 14 of incubation in riboflavin-deficient embryos. This impairment of MCAD and other flavin-dependent enzymes leads to a metabolic block that results in the observed accumulation of lipid in the embryonic liver, a dramatic 30-fold increase in 10, 12, and 14-carbon intermediates of fatty acid oxidation in liver, and the inability to synthesize polyunsaturated fatty acids [16].

With fatty acid oxidation severely inhibited, it appears that riboflavin-deficient embryos depend heavily on glycolysis for ATP production. Plasma glucose concentrations that remain relatively unchanged or even rise slightly in normal embryos between day 10 and 14 of incubation, rapidly drop to near zero at the time of death in Rf- embryos [16]. At the same time, lactate concentrations in allantoic fluid, an indicator of carbohydrate catabolism, remain higher than 2-hydroxybutyrate, a ketone body produced during lipid catabolism. In normal embryos and riboflavin-deficient embryos supplemented with riboflavin, the concentration of 2-hydroxy-butyrate is always higher than lactate in allantoic fluid. A progressive decrease in heart rate in riboflavin-deficient embryos [in a tissue with increasing demand for fatty acid oxidation] [17] begins about 1 hour before death suggesting that the rate of ATP production from lipid and carbohydrate sources is insufficient to sustain cardiac muscle contraction and leads to death [16]. Thus, the sudden death of all riboflavin-deficient chicken embryos results from a cascade of catastrophic events precipitated by depletion of trace amounts of riboflavin that sustain the minimal demands of early embryo growth, but become limiting after embryonic day 11 (e11) of the 21-day incubation period. Initially, riboflavin deficiency inhibits fatty acid oxidation, the major energy source for avian embryos, and causes excessive accumulation of metabolic intermediates that sequester Coenzyme A (CoA), which in turn has further inhibitory effects on pathways generating ATP. The increased dependence on glycolysis soon depletes the *rd/rd* embryo’s limited reserves of glucose, and then death soon ensues. In humans, hereditary medium-chain acyl-coenzyme A dehydrogenase disorder (MCADD) can lead to sudden infant death due to the decreased ability to metabolize lipids during an overnight fast [18]. The crisis in lipid oxidation induced by acute riboflavin deficiency in chicken embryos metabolically mimics the sudden infant death syndrome due to MCADD. Several distinct tissues express three major transporters for riboflavin in mammals: the placenta, intestine and kidney (RFVT1), the brain (RFVT2), and visceral glands/organs (RFVT3) [19]. In contrast, birds are oviparous animals, which require the dam to have a specialized system, the riboflavin binding protein (RBP), to transport riboflavin (Rf) from the post-prandial liver to the oviduct for deposition of essential riboflavin into the egg prior to oviposition, incubation and embryonation. Consequently, RBP is the highest expressed gene found in liver of the laying hen, being 67-fold greater than in liver of non-laying chickens, using the same 20.7 K chicken oligo microarrays used in the present study [20].

Here, we describe transcriptional changes in liver of riboflavin-deficient chicken embryos, with or without riboflavin supplementation, between embryonic day e9 and e15. Our goal was to identify riboflavin-specific responses by comparing riboflavin -rescued (Rf+) *verses* riboflavin-deficient (Rf-) embryos during this abrupt metabolic collapse. Our unique single-nutrient (Rf) model allowed the identification of riboflavin-dependent hepatic genes and their functional networks involved in the switch to alternative energy sources and global adaptation to acute riboflavin deficiency, which leads to sudden embryo death just before or on e15. Our study provides an excellent model of the maternal effect, where the mother’s genotype (*rd/rd*) determines the survival of embryos, whereas the embryo’s genotype (*rd/rd*) has little effect.

## Methods

### Experimental birds and riboflavin treatment

Single-Comb White Leghorn (SCWL) chickens, homozygous recessive for the riboflavin binding-protein deficient-allele (*rd/rd*) were maintained at the University of Delaware. The chickens were raised and cared for under protocols approved by the Institutional Animal Care and Use Committee (ICUC), University of Delaware. Fertile eggs, derived from riboflavin binding protein deficient (*rd/rd*) hens, were injected with riboflavin-5′-phosphate (FMN) [riboflavin-rescued (Rf+)] or sterile saline [riboflavin-deficient (Rf-)] at the onset of incubation (e0). A total of 150 fertile eggs were collected from *rd/rd* hens and one-third of the eggs were each injected with 100 μL of FMN (540 μg) just before onset of incubation (e0) and assigned to the riboflavin-rescued (Rf+) treatment. The FMN (540 mg) was dissolved in 100 mL of sterile normal (0.85 M) saline, the solution was then filter sterilized and stored in darkness at 4 °C until usage. The remaining two-thirds of the eggs were injected with 100 μL of sterile saline; these eggs were assigned to the riboflavin-deficient (Rf-) treatment. The eggs were then incubated in an automated Jamesway Egg Incubator (Cambridge, ON, CDN) maintained at 39 °C and 95% relative humidity (RH). A larger number of fertile riboflavin-deficient eggs were incubated to compensate for very high mortality found in Rf- embryos at e13 and e15, since riboflavin deficiency was 100% lethal after e15 [21].

### Liver sampling and RNA extraction

Eight viable embryos from each riboflavin treatment group were killed for liver samples at two embryonic ages (e9 and e11), while four individual liver samples were collected from each group at e13, and e15 (Table 1). Due to the very small size of liver in embryos at e9 and e11, two livers from each riboflavin treatment group (Rf + and Rf-) were randomly selected and pooled in pairs to represent each of the four biological replicates/riboflavin treatment used for RNA extraction at e9 and e11. Liver samples were immediately placed in 2 ml Corning® cryogenic vials, snap frozen in liquid nitrogen and stored frozen at − 80 °C until processing for RNA. Thus, a total of 32 liver samples represents the Rf + and Rf- treatments at four embryonic ages (e9, e11, e13, and e15), with four biological replicates for each riboflavin treatment x age combination. Gross anatomy of Rf + and Rf- embryos at e15 was documented using a Leica M420 macroscope (Buffalo Grove, IL) fitted with a Spot Insight 4 imaging system; images were taken at a magnification of 10X.

**Table 1:** Experimental Design

Embryonic age, day (e)	Riboflavin-rescued (Rf+) [n]	Riboflavin-deficient (Rf-) [n]
e9	8 (4 biological replicates)	8 (4 biological replicates)
e11	8 (4 biological replicates)	8 (4 biological replicates)
e13	4	4
e15	4	4

Fertile eggs, derived from riboflavin binding protein deficient (*rd/rd*) Single-comb White Leghorn (SCWL) chickens, were injected with riboflavin 5 phosphate (FMN) [riboflavin-rescued (Rf+)] or saline [riboflavin-deficient (Rf-)] at the onset of incubation (e0). Eight viable embryos were killed for liver samples at e9 and e11, while four viable embryos were killed for liver samples at e13 and e15. Two liver samples were randomly pooled to provide 4 biological replicates for each treatment group at e9 and e11, while 4 individual livers samples represent biological replicates for each treatment group at e13 and e15. The very small size of the liver in e9 and e11 required pooling of 2 livers to create each of the 4 biological replicates per riboflavin treatment group for the first two ages (e9 and e11). Total RNA was purified from these 32 liver samples and used for both microarray analysis and qRT-PCR analysis

Total RNA was extracted from each frozen liver sample using a RNeasy Mini kit (Qiagen, Valencia, CA), following the Qiagen protocol for the extraction of total cellular RNA with minor modifications. The quantity of the RNA was determined using a NanoDrop spectrophotometer (Wilmington, DE). RNA quality was analyzed by microcapillary electrophoresis on an Agilent Bioanalyzer 2100 (Agilent Technologies, Wilmington, DE), where the ribosomal RNA ratio (28S/18S) was assessed for RNA integrity. The RNA was diluted to a standard concentration (250 ng/μL). Liver RNA samples (1 μL each) were loaded onto a RNA 6000 Nano Chip and analyzed. RNA with a RNA Integrity Number (RIN) between 9 and 10 indicated high quality and was used for amplification of RNA.

### RNA amplification and dye labeling

RNA amplification was performed with an Amino Allyl MessageAmp™ II aRNA Amplification kit according to the manufacturer’s protocol (Ambion; Austin, TX). Briefly, a single round amplification of RNA was completed using 4 μg of total RNA. The in vitro transcription (IVT) reaction of amino allyl-modified RNA (aRNA) was incubated for 14 h at 37 °C. The aRNA was recovered, determined qualitatively with a RNA 6000 Nano Assay kit, and quantitatively with a NanoDrop ND-1000 spectrophotometer prior to the aRNA/dye coupling reaction. Twenty microgram of aRNA was dried down to a minimal volume in a Savant Speed Vac, reconstituted in 9 μL of coupling buffer and mixed with 11 μL of prepared dye [i.e., a 60 μg aliquot of either Alexa Fluor® 555 or Alexa Fluor® 647 dye (Molecular Probes, Inc. Eugene, OR) dissolved in DMSO]. A reference pool was made from an equal quantity of aRNA from each of the 32 biological samples and multiple aliquots of reference aRNA labeled with Alexa Fluor® 647. The 32 aRNA samples, representing two riboflavin treatment groups at 4 embryonic ages (Table 1), were individually labeled with Alexa Fluor® 555. Each labeled target and reference aRNA sample was then purified on a spin column, analyzed with a NanoDrop ND-1000 spectrophotometer, and dye incorporation determined.

### Oligo microarrays and hybridization

The chicken long oligos were designed by ARK-Genomics [22] from multiple chicken transcript databases. Chicken expressed sequence tags (ESTs) and transcript sequences were aligned and 70mer oligos designed, using OligoArray2.0, against regions common to more than one aligned sequence. The chicken oligo set [*Gallus gallus* (chicken) Roslin/ARK CoRe Array V1.0] was manufactured by Operon Technologies, Inc. (Alameda, CA). This chicken oligo set (Catalog # 851102) contains 20,460 long oligo probes (300 pmol) arrayed in 54,384-well plates. For quality control, 4 wells in each of the 54,384-well plates contained the Operon Production Tracking Oligo opHsV04NC000001, which is a randomly-generated oligo sequence with a length of 30 bases.

The “Arizona” 20.7 K chicken oligo arrays were printed in a horizontal pattern by the Genomics Research Laboratory, Steele Children’s Research Center, University of Arizona, (Tucson AZ). Each Arizona 20.7 K chicken oligo array, (NCBI GEO Platform GPL6049) [23], was composed of 20,676 features that include 20,460 70mer oligos and 216 control spots of the Operon Production Tracking Oligo opHsV04NC000001. The oligos (300 pmol) were dissolved in 8 μL of 50% DMSO for printing. The chicken oligo array was printed in a horizontal pattern onto Corning® UltraGAPSTM aminosilane coated slides (Cat # 40018) using a Virtek ChipWriter Pro (Model # HCO1-M) robot fitted with 48 TeleChem (SMP2.5) pins in a HEPA-filtered positive-pressure chamber maintained at 24 °C and 48% RH (± 2 %RH). The printed slides were dried overnight at room temperature (22-25 °C with 20-40% RH). The slides were then stored in a chemical (Drierite) desiccator at room temperature until use.

The oligo microarrays were baked at 90 °C for 1 hour prior to use. The arrays were pre-treated for 45 min at 42 °C in pre-hybridization solution containing 5× SSC, 0.1% SDS and 1% BSA and followed by dipping for 5 min in 2× SSC and then 0.2× SSC. The slides were spin dried for 5 min at 1000 x g. Eight microgram of labeled aRNA was fragmented in 1× fragmentation buffer (Ambion) for 15 min at 70 °C. The reaction was then terminated by adding stop solution and dried down (SpeedVac) to a minimal volume. Each labeled target and reference RNA sample was reconstituted prior to hybridization in a 30 μL volume of pre-warmed DIG Easy Hyb solution (Roche Diagnostics Corporation; Indianapolis, IN). All reference samples were pooled together and aliquot into 30 μL. An equal amount (8 μg) of target and reference samples were mixed with 2.5 μL of 10 mg/ml yeast tRNA, 2.5 μL of 10 mg/ml salmon testes DNA (Sigma, Louis, MO). The reference/target mixture (65 μL) was denatured for 2 min at 94 °C, cooled at room temperature, carefully loaded onto the middle of a pre-treated microarray slide held in a hybridization chamber (Corning #2551, Lowell, MA) and overlaid with a 22 × 65 mm LifterSlip (Erie Scientific, Portsmouth, NH). The sealed hybridization chamber was incubated overnight (14-16 h) at 42 °C in a water bath under a light-tight box. On the following day, slides were sequentially washed with 1 x SSC, 0.2% SDS, and 0.5% DTT at 42 °C for 10 min, then 0.1 x SSC, 0.2% SDS, and 0.5% DTT for 5 min at room temperature, and finally 0.1 x SSC and 0.5% DTT for 1 min at room temperature (with high-purity nitrogen gas bubbled through each of the wash solutions to prevent interference by ozone). The slides were subsequently rinsed in distilled water and dried by centrifugation. Before scanning, slides were stored in individual 50 ml tubes covered in aluminum foil and filled with nitrogen gas.

### Microarray data analysis

The 32 amplified liver RNA samples were hybridized to 32 chicken long-oligo microarrays using a reference RNA design and blocked on hybridization day (Day 1 or Day 2); where, 32 microarray slides represented 4 biological replicates across 2 riboflavin treatments and 4 embryonic ages (Additional file 1). Liver amplified RNA samples were labelled with Alexa Fluor™ 555 dye and an aliquot of the reference RNA pool was labelled with Alexa Fluor™ 647 dye. A GenePix 4000B scanner with GenePix Pro 4.1 software (Molecular Devices, Union City, CA) was used to scan hybridized slides at wavelengths of 532 nm for the Alexa Fluor™ 555-label and 635 nm for the Alexa Fluor™ 647-label. The scanner laser power was set at 100% and the photomultiplier tube (PMT) setting was adjusted to unity for each scan. The GenePix software provided a combined TIFF image file for each slide. The high-resolution image files were first analyzed using GenePix Pro v4.1 software, without correction for background. The GenePix report (gpr) files were automatically merged with an annotated Excel file containing the oligo identification [Roslin Institute *Gallus gallus* (RIGG)] number and array metadata [24] on our laboratory website [25] using the “Oligo Arrays” function designed for the Arizona 20.7 K Chicken Oligo Array (v2).

### Normalization and statistical analysis of microarray data

Limma software was used for processing, normalization and determination of differential gene expression [26]. Median intensities for each dye were Loess normalized (without background subtraction) within array and between array to correct for dye and slide biases. The Benjamini-Hochberg procedure [27] was used to control the experiment-wise false discovery rate (FDR ≤ 0.05) due to multiple testing procedures. Gene expression represents the log2 ratio of normalized dye ratios (Experimental aRNA/Reference aRNA). A gene was considered differentially expressed (DE) if the log2 fold-change (Alexa Fluor™ 555/Alexa Fluor™ 647 intensity ratio) was significant as indicated by the adjusted *p*-value (*P* ≤ 0.05) and FDR ≤ 0.05. The Limma output data were then statistically analyzed using a linear mixed model in the Statistical Analysis System (SAS; Cary NC) [28, 29], which we have described earlier [30, 31]. Mixed models were used to account for biological and technical variation (dye, day of hybridization) across the 32 oligo microarrays and to determine the main effects of riboflavin treatment (Rf + *verses* Rf-) and embryonic age, and their interaction. The DE gene lists derived from statistical analyses were then submitted to Ingenuity® Pathway Analysis for final annotation and functional analysis.

### Ingenuity Pathway Analysis and Ingenuity Up-Stream Regulator Analysis

The DE gene lists generated from statistical contrast were used as input files for annotation and functional analysis using Ingenuity® Pathway Analysis (IPA) [32]. Only DE genes with a NCBI Reference Sequence (Ref-Seq) Protein ID are accepted IPA as “Analysis Ready” (AR) and imported into the online database for a “Core Analysis”, which maps AR-DE genes to canonical pathways, biological processes and gene interaction networks accrued in the Ingenuity® Knowledge Base, which is largely mammalian concentric and devoid of annotated avian-specific genes. The Ingenuity® Up-stream Regulator Analysis was used to identify major transcription factors, direct interaction with other upstream regulators, and direct interactions with target genes to predict activation or inhibition of particular biological pathways and processes. IPA uses the Fisher’s Exact Test to provide significance (*P* ≤ 0.05) of over-representation of AR-DE genes in canonical pathways and biological processes, based on the probability that AR-DE genes are associated with a particular biological process or pathway.

### Quantitative RT-PCR (qRT-PCR) analysis and verification of differential gene expression

Quantitative reverse transcriptase polymerase chain reaction (qRT-PCR) analysis was performed to verify select gene expression patterns found by microarray analysis. A total of 42 primers were designed using Primer Express v2.0 (Applied Biosystems, Foster City, CA). Details of primer design including the Roslin Institute *Gallus gallus* (RIGG) oligo ID number, gene symbol, forward and reverse sequences, and amplicon size (bp) are provided (Additional file 2). First-strand cDNA synthesis was completed using Superscript III reverse transcriptase (Invitrogen), an oligo (dT) primer, and 1 μg of purified total RNA. The qRT-PCR analysis was performed in 384-well plates containing 1 μL of cDNA template/well and 5 μL of a super mix providing: 3 μL SYBR® Green PCR Master Mix, 1.5 μL RNase-DNase free water and 0.5 μL of each primer (concentration 10 μM). A total of eight plates were analyzed in an ABI Prism Sequence Detection System (Model 7900HT), where the 384-well plate format allowed duplicate wells for four primer sets across the 24 candidate genes and a panel of 6 invariant “housekeeping” genes, which were used for evaluation and normalization of qRT-PCR expression data. The average cycle time (Ct) for each biological sample was normalized to the geometric mean of the most stable “housekeeping” genes using Biogazelle qbase+ [33] software. The most invariant genes (*COX7A2L* and *PCF11*) identified by the geNorm software were used for normalization and calculating relative transcript abundance, which was based on the 2^ΔΔCt^ formula [34]. The normalized qRT-PCR gene expression values from this factorial design experiment [2 riboflavin treatments × 4 embryonic ages (e9-e15)] were then analyzed by two-way analysis-of-variance (ANOVA) using the general linear model (GLM) procedure in the Statistical Analysis System (SAS; v9.3.1). The GLM procedure allowed determination of the significant (*P* ≤ 0.05) effects of riboflavin treatment, age, and their interaction. The protected Fisher’s least significant difference (LSD) test was used for mean separation. The Pearson’s Correlation Analysis was completed using 21 DE “candidate” genes identified by microarray analysis and the normalized expression levels of these genes obtained by the qRT-PCR analysis described above.

An additional qRT-PCR analysis was completed on 12 additional genes of special interest. The abundance of 12 additional genes was determined on an identical set of 32 RNA samples representing 4 biological samples/treatment (Rf + and Rf-) at 4 embryonic ages (e9, e11, e13 and e15). Relative transcript abundance was based on the 2^ΔΔCt^ formula [34] and normalized against a single invariant gene (*COX7A2L*). These expression levels are presented as arbitrary units (AU) and represent the mean ± SEM of four embryos/riboflavin treatment across 4 ages. The Student’s *t*-test was used to determine significant (*P* ≤ 0.05) differences between Rf + and Rf- embryos at each age.

## Results

### Gross anatomical features of riboflavin-deficient and riboflavin-rescued chick embryos

Figure 1a shows the gross anatomy of typical riboflavin-deficient (Rf-) and riboflavin-rescued (Rf+) chick embryos at e15. The mortality of riboflavin-deficient embryos increased sharply between e13 and e15, reaching more than 75% by e15. The photo-macrographs show the head of typical *rd/rd* embryos, with (Rf+) and without (Rf-) riboflavin supplementation, immediately after their removal alive from the eggshell. Notable hallmarks of riboflavin-deficiency in *rd/rd* chick embryos were retarded development of the head, eye, beak and feathers, and extensive cutaneous hemorrhage (Fig. 1a). The liver of the Rf- embryo shows massive hemorrhage and engorgement with yolk lipids along its perimeter. In contrast, the head of the Rf + embryo shows normal development of the beak, head, eye and feathers. Likewise, the Rf + embryo presents with a pale yellow-colored liver, indicating normal lipid accumulation and utilization of a normal chick embryo at e15.

### Differentially expressed genes identified by microarray analysis

There were no differentially expressed genes found between Rf- and Rf + embryos at e9 and e11. Therefore, only the main effect of riboflavin treatment (at e13 and e15) was considered for further functional analyses using IPA. In contrast to the lack of DE genes at e9 and e11, 396 differentially expressed (DE; adjusted *P* ≤ 0.05 and FDR ≤ 0.05) genes were identified in e13 embryos; while 1467 DE genes were found between Rf- and Rf + embryos at e15 (Additional file 3). [It should be noted that the DE genes indicated above represent all DE oligo spots identified by statistical analysis, whether or not the RIGG oligo was annotated.] The reduced number of DE hepatic genes accepted by IPA as “Analysis Ready” was due to the lack of an annotation [NCBI Ref-Seq protein ID] for 74 “unknown” RIGG oligos at e13. Likewise, another 248 DE “unknown” RIGG oligos identified at e15 were rejected by IPA from further functional analyses. The last annotation of the chicken 20.7 K RIGG oligo array was published in 2009 [24] with 23% of the RIGG oligo IDs void of any annotation (i.e., unknown). Accordingly, this is similar to our observed rejection of 17% of DE RIGG oligos (248 genes) from our microarray analysis of liver across both riboflavin-deficient (98 RIGG IDs rejected) and -rescued (149 RIGG IDs rejected) embryos without a functional analysis by IPA. Consequently, only 221 DE genes at e13 and 929 DE genes at e15 were matched to annotated genes accrued in the Ingenuity® Knowledge Base (Fig. 1b). Genes mapped to NCBI Entrez IDs or Ref-Seq proteins accrued in the Ingenuity® Knowledge Base are considered by IPA as “Analysis Ready” and denoted as AR-DE genes in subsequent figures and tables. There were 59 unique AR-DE genes at e13 and 767 unique AR-DE genes, whereas 162 AR-DE genes were commonly shared between e13 and e15 embryos. The e13 and e15 AR-DE gene sets were functionally annotated using the Core Analysis feature in IPA software and the Ingenuity® Upstream Regulator Analysis.

### Ingenuity Pathway Analysis of AR-DE genes in liver of Rf + and Rf- embryos at e13

A summary of the IPA analysis of e13 embryos is presented in Table 2. The top five canonical pathways identified in liver of e13 embryos were “IL-8 Signaling, JAK/Stat Signaling, EIF2 Signaling, RANK Signaling in Osteoclasts, and Proline Biosynthesis I”. Ingenuity Upstream Regulators Analysis predicts that E2F transcription factor 1 (E2F1), MYC proto-oncogene, bHLH transcription factor (MYC), activating transcription factor 4 (ATF4), transcription factor 3 (TCF3) and peroxisome proliferator activated receptor alpha (PPARA) were the major transcriptional regulators of AR-DE genes found at e13. The “Top Molecular and Cellular Functions” identified by IPA at e13 were “Amino Acid Metabolism, Small Molecule Biochemistry, Molecular Transport, Cell Cycle and Cell Death/Survival”. The major divisions of the “Physiological System Development and Function” category were related to function and development of the endocrine, cardiovascular and digestive systems, whole organism and organ morphology. Another category identified by IPA was “Hepatotoxicity”, which included several subdivisions (“Liver Proliferation, Steatosis, Fibrosis, Carcinoma, and Hyperplasia/Hyper-proliferation”). Among the top 10 genes that were expressed higher in the liver of Rf + embryos at e13 were 3 coagulation factors [thrombin (*F2*), vitamin K-dependent coagulation factor IX (*F9*), and vitamin K-dependent protein Z (*PROZ*). The top 10 over-expressed genes in the liver of Rf- embryos at e13 were of a higher magnitude (log2-1.4 to − 2.7) than those of the Rf + embryos and included ankyrin repeat domain 22 (*ANKRD22*), ovochymase 2 (*OVCH2*), aldehyde dehydrogenase 18 family member A1 (*ALDH18A1*), carbonic anhydrase 2 (*CA2*), galectin 1 (*LGALS1*), retinol binding protein 7 (*RBP7*), pyrroline-5-carboxylate reductase family member 2 (*PYCR2*), perilipin 2 (*PLIN2;* which coats intracellular lipid droplets), and asparagine synthetase, glutamine-hydrolyzing *(ASNS).* Interestingly, four of these highly expressed hepatic genes found in Rf- embryos are involved in synthesis of several amino acids (*ALDH18A1, LGALS1, PYCR2* and *ASNS*).

**Table 2:** IPA summary of microarray analysis of liver in riboflavin-deficient chick embryos at e13

Top Canonical Pathways	*p*-value	Overlap	Ratio
IL-8 Signaling	3.85E-05	5.1%	10/197
JAK/Stat Signaling	2.14E-04	7.2%	6/83
EIF2 Signaling	4.85E-04	4.1%	9/221
RANK Signaling in Osteoclasts	6.17E-04	5.9%	6/101
Proline Biosynthesis I	6.22E-04	50.0%	2/4
Top Upstream Regulators	*p*-value of overlap		# Target genes
E2F1	3.40E-07		19
MYC	5.14E-07		30
ATF4	1.60E-06		10
TCF3	4.20E-06		12
PPARA	9.09E-06		16
Top Molecular and Cellular Functions	*p*-value		# Genes
Amino Acid Metabolism	1.03E-02 - 1.07E-07		22
Small Molecule Biochemistry	1.03E-02 - 1.07E-07		58
Molecular Transport	1.03E-02 - 5.02E-06		59
Cell Cycle	1.03E-02 - 8.67E-06		38
Cell Death and Survival	1.03E-02 - 5.35E-05		68
Physiological System Development and Function	*p*-value		# Genes
Endocrine System Development and Function	1.03E-02 - 5.02E-06		7
Organismal Development	1.03E-02 - 9.26E-06		64
Cardiovascular System Development and Function	8.81E-03 - 1.41E-05		31
Organ Morphology	1.03E-02 - 1.41E-05		40
Digestive System Development and Function	9.14E-03 - 1.74E-05		34
Hepatotoxicity	*p*-value		# Genes
Liver Proliferation	2.04E-02 - 8.34E-05		10
Liver Steatosis	4.45E-01 - 1.55E-03		11
Liver Fibrosis	3.91E-01 - 1.72E-03		9
Hepatocellular Carcinoma	2.28E-01 - 2.89E-03		19
Liver Hyperplasia/Hyper-proliferation	2.43E-01 - 2.89E-03		76
Top Up-regulated genes	log2 Rf+/Rf-	Top Down-regulated genes	log2 Rf+/Rf-
*CHODL*	1.41	*ANKRD22*	−2.74
*FAM84A*	1.27	*OVCH2*	−2.42
*PROZ*	1.24	*EIF5B*	−2.01
*TOP1*	1.21	*ALDH18A1*	−1.99
*COMT*	1.18	*CA2*	−1.97
*F9*	1.17	*LGALS1*	−1.77
*RUFY2*	1.17	*RBP7*	−1.75
*GATM*	1.14	*PYCR2*	−1.68
*F2*	1.09	*PLIN2*	−1.67
*GC*	1.07	*ASNS*	− 1.38

Ingenuity Pathway Analysis (IPA) of 221 “Analysis Ready” (AR) and differentially expressed (DE; FDR ≤ 0.05) (AR-DE) genes from riboflavin-rescued (Rf+) and riboflavin-deficient (Rf-) chickens on embryonic day 13 (e13). *P*-values were determined by IPA using Fisher’s Exact Test and represent the likelihood that AR-DE genes are over-represented and associated with a particular biological process or pathway. The percent overlap and ratios were calculated for the number of observed AR genes compared to the number of known genes for that category represented in the Ingenuity® Knowledge Base

### Ingenuity Pathway Analysis of AR-DE genes in liver of Rf + and Rf- embryos at e15

The summary of IPA analysis of the 929 AR-DE genes found in e15 embryos is presented in Table 3. The top five canonical pathways populated by the AR-DE genes on e15 were “EIF2 Signaling, Intrinsic Prothrombin Activation, LXR/RXR Activation, Tryptophan Degradation, and Cholesterol Biosynthesis”. The “Top Molecular and Cellular Functions” over-represented by AR-DE genes were “Protein Synthesis” (139 genes), “Lipid Metabolism” (173 genes), “Small Molecule Biochemistry” (212 genes), “Amino Acid Metabolism” (47 genes), and “Vitamin and Mineral Metabolism” (61 genes). The top five categories under “Physiological System Development and Function” were “Tissue Morphology, Organismal Development, Connective Tissue Development and Function, Tissue Development, and Digestive System Development and Function”. The major sub-categories and direct target genes under “Hepatotoxicity” were “Hepatocellular Carcinoma” (64 genes), “Liver Hyperplasia/Hyperproliferation” (313 genes), “Liver Fibrosis” (23 genes), “Liver Proliferation” (24 genes), and “Liver Necrosis/Cell Death” (20 genes). The 10 most highly-expressed genes found in the liver of Rf + embryos at e15 were feather keratin (*FKER*), alpha 2-HS glycoprotein (*AHSG*), serine peptidase inhibitor, Kazal *type* 5 (*SPINK5;* an inhibitor of thrombin*),* albumin (*ALB*), serpin family C member 1 (*SERPINC1*), angiopoietin like 3 (*ANGPTL3*), plasminogen (*PLG*), glycine amidinotransferase (*GATM*) and nebulette (*NEBL*). Among the 10 highest expressed genes in liver of Rf- embryos at e15 were 3-hydroxy-3-methylglutaryl-CoA synthase 1 (*HMGCS1*), ankyrin repeat domain 22 (*ANKRD22*), ovochymase 2 (*OVCH2*), 3-hydroxy-3-methylglutaryl-CoA synthase 1 (*HMGCL*), 3-hydroxymethyl-3-methylglutaryl-CoA lyase (*HMGCL*), *LGALS, RBP7,* and acetyl-CoA acetyltransferase 2 (*ACAT2*), which are involved in lipid metabolism.

**Table 3:** IPA summary of microarray analysis of liver in riboflavin-deficient chick embryos at e15

Top Canonical Pathways	*p*-value	Overlap	Ratio
EIF2 Signaling	9.53E-08	13.1%	29/221
Intrinsic Prothrombin Activation Pathway	1.99E-07	34.5%	10/29
LXR/RXR Activation	2.25E-07	16.5%	20/121
Tryptophan Degradation III	3.58E-07	37.5%	9/24
Super-pathway of Cholesterol Biosynthesis	1.62E-06	32.1%	9/28
Top Upstream Regulators	*p*-value of overlap		# Target genes
HNF4A	4.81E-14		161
MYC	5.70E-11		93
PPARA	1.34E-10		50
ESR1	5.09E-10		150
NFE2L2	9.70E-08		105
Top Molecular and Cellular Functions	*p*-value		# Genes
Protein Synthesis	2.61E-03 - 8.55E-11		139
Lipid Metabolism	3.08E-03 - 7.82E-10		173
Small Molecule Biochemistry	3.33E-03 - 7.82E-10		212
Amino Acid Metabolism	3.19E-03 - 4.41E-09		47
Vitamin and Mineral Metabolism	2.18E-03 - 7.36E-09		61
Physiological System Development and Function	*p*-value		# Genes
Tissue Morphology	3.00E-03 - 1.44E-06		131
Organismal Development	3.31E-03 - 1.84E-06		246
Connective Tissue Development and Function	2.92E-03 - 5.93E-06		158
Tissue Development	3.31E-03 - 5.93E-06		159
Digestive System Development and Function	1.85E-03 - 7.57E-06		77
Hepatotoxicity	*p*-value		# Genes
Hepatocellular Carcinoma	6.04E-01 - 9.22E-05		64
Liver Hyperplasia/Hyperproliferation	6.04E-01 - 9.22E-05		313
Liver Fibrosis	4.36E-01 - 2.50E-04		23
Liver Proliferation	4.60E-01 - 2.50E-04		24
Liver Necrosis/Cell Death	4.04E-01 - 1.85E-03		20
Top Up-regulated genes	log2 Rf+/Rf-	Top Down-regulated genes	log2 Rf+/Rf-
*FKER*^a^	3.43	*HMGCS1*	−3.61
*AHSG*	2.57	*OVCH2*	−2.61
*SPINK5*	2.38	*ANKRD22*	−2.58
*ALB*	2.27	*HMGCL*	−2.40
*SLC13A3*	2.21	*LGALS1*	−2.34
*SERPINC1*	2.17	*RBP7*	−2.09
*ANGPTL3*	2.14	*ACAT2*	−1.99
*PLG*	2.10	*TPPP*	−1.92
*GATM*	2.06	*EIF5B*	−1.78
*NEBL*	2.06	*RPL22L1*	−1.78

Ingenuity Pathway Analysis (IPA) was used for functional analysis of 929 “Analysis Ready” (AR) and differentially expressed (DE; FDR ≤ 0.05) (AR-DE) genes from riboflavin-rescued (Rf+) and riboflavin-deficient (Rf-) chickens on embryonic day 15 (e15)

^a^Note: *FKER* was the highest expressed gene found in liver of Rf + embryos at e15 (see Additional file 3, Riboflavin_e15 worksheet). However, this avian-specific gene was not annotated in the Ingenuity® Knowledge Base and therefore rejected by IPA

Detailed information on AR-DE genes that populate major canonical pathways is provided in Additional file 4. The eukaryotic translation initiation factor 2 (EIF2) Signaling Pathway contained 29 AR-DE genes (see Table 3); and of these, only 3 AR-DE genes (*ATF4, CCND1* and *BCL2*) were expressed higher in the liver of Rf + embryos whereas, 26 AR-DE genes (eukaryotic translation initiation factors and ribosomal proteins) genes were over-expressed in liver of Rf- embryos at e15. In contrast, 19 of the 20 AR-DE genes found in the LXR-RXR Activation Pathway, that controls lipid metabolism, were more abundant in liver of Rf + embryos than Rf- embryos, which had only a single up-regulated gene (*CYP51A1*), which controls cholesterol biosynthesis. Farnesoid X receptor (FXR)-RXR Activation is another canonical pathway predominately populated by 17 AR-DE genes, including *AHSG, ALB, APOH* and *GC* (or vitamin D binding protein), that were expressed higher in the Rf + embryos, compared to only a single gene (*PPARG*) that was over-expressed in liver of Rf- embryos. Acute phase response signaling, which provides a rapid non-specific inflammatory response against infection, was also dominated by 17 AR-DE genes up-regulated in the Rf + embryo, compared to only 5 genes that were expressed higher in liver of the Rf- embryos.

The “Intrinsic Prothrombin Activation” pathway in IPA was over-represented by 10 AR-DE genes (*SERPINC, F2, FGB, FGA, FGG, F9, COL3A1, COL2A1, PROC* and *F13B*), which were highly expressed in the Rf + embryos at e15. The IPA Blood Clotting mechanism was over-represented by 23 highly-expressed AR-DE genes in Rf + embryo whereas only two genes (*PLA2G4A* and *LGALS1*) were over expressed in liver of Rf- embryos at e15. The highly expressed hemostasis genes found in liver of the Rf + embryos include proteases (*CPB2, F2, F9, PLG*) protease inhibitors (*AGT*, *SERPINC1*), collagen (*COL14A1*), clotting factors (*FGA, FGB, FGC, F13B*) and cofactors (*APOH*, *PROC*, *PROCZ*). Several of these clotting genes (*ALB, AGT, APOH, PLG, F2, FGA, FGB* and *FGG*) are also involved in “Acute Phase Response Signaling”.

“Fatty Acid Metabolism”, a sub-category under “Lipid Metabolism” in IPA (Table 3) was over-represented by 66 AR-DE genes, where 45 AR-DE genes were highly expressed in the Rf + liver and only 21 genes were expressed at higher levels in the Rf- embryos (Additional file 5). Another sub-category under Lipid Metabolism was “Oxidation of Lipid”, which included 16 AR-DE genes that were more abundant in the Rf- embryos, compared to 7 genes expressed higher in the liver of Rf- embryos. Also of interest was Carbohydrate (CHO) Metabolism, which was also overrepresented by 37 highly-expressed AR-DE genes found in the Rf + embryos, whereas only 19 genes associated with CHO metabolism were expressed higher in liver of Rf- embryos. IPA assigned 60 AR-DE genes to “Protein Catabolism”; and of these, 39 genes were expressed at higher levels in the Rf + embryo, while only 22 genes were associated with catabolism of protein in the Rf- embryo, which have a more active proteasome than do the Rf + embryos at e15. “Metabolism of Amino Acids” (AA) was another functional category of particular interest, where 16 AR-DE genes were upregulated in the Rf + livers while 13 genes were upregulated in the Rf- embryos. Of the 20 AR-DE genes functionally annotated by IPA as “Protein Ubiquitination”, only two genes were expressed higher in liver of the Rf + embryos, whereas 18 genes were expressed higher in the Rf- embryos.

Twelve AR-DE genes were assigned to metabolism of vitamins with six genes upregulated in each riboflavin treatment group. Furthermore, we mapped 95 homologs of human flavoproteins onto the chicken genome sequence. And of these, 15 flavoprotein genes were expressed higher in liver of Rf + at e15, which indicates recovery; whereas only four of the DE genes were over-expressed in liver of Rf- embryos at e15, indicating global riboflavin deficiency.

### Gene interaction networks generated from liver of Rf + and Rf- embryos at e13 and e15

**Fig. 2:**
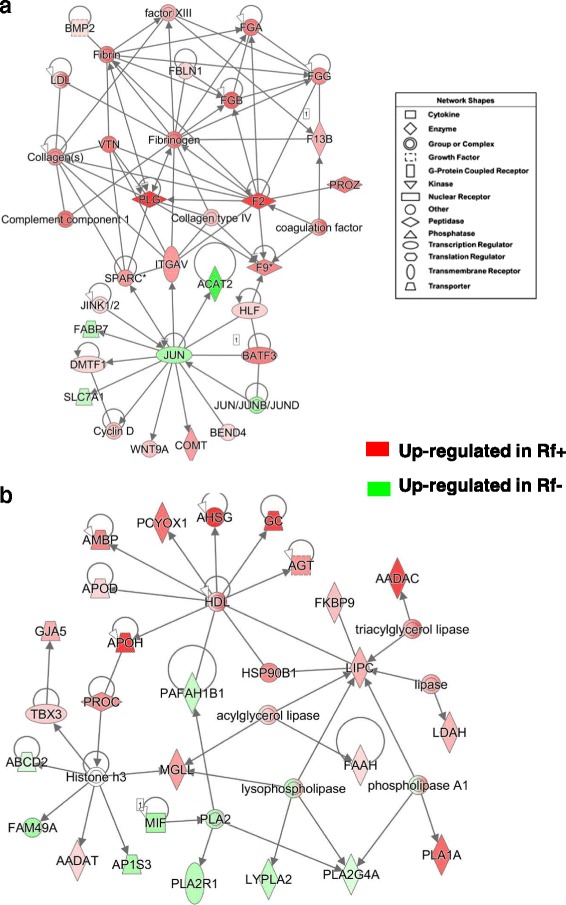
Two direct gene interaction networks identified by IPA in liver of Rf + and Rf- embryos at e15. The top panel (**a**) shows a gene interaction network functionally annotated by IPA as involved in “Hematological Disease, Organismal Injury and Abnormalities”. The bottom panel (**b**) shows a gene interaction network related to “Lipid Metabolism” and “Molecular Transport”. The legend indicates the major functional class of each gene. Genes with red-colored symbols are expressed higher in liver of Rf + embryos at e15, whereas green-colored symbols indicate higher expression in liver of Rf- embryos at e15

IPA annotated the gene interaction network depicted in Fig. 2a as related to “Organismal Injury and Abnormalities, Hematological Disease”. Numerous coagulation factors are overexpressed in the liver of Rf + embryos at e15 as indicated in Table 3 and Additional file 4 (*F2, F8, F13B, FGA, FGB, FGG, PROC, PROZ, PLG*, and *VTN*)*.* A more comprehensive list of DE (*P* ≤ 0.10) coagulation genes is provided in Table 4, which shows 25 DE genes that belong to “Blood Clotting” in the “BioFunction” category of IPA. Some of these coagulation factors directly interact with genes (*SPARC*, *ITGAV* and *HLF*) that are direct targets of Jun proto-oncogene, AP-1 transcription factor subunit (*JUN*), which was up regulated in liver of the Rf- embryo at e15. Most of these coagulation factors are part of the “Intrinsic Prothrombin Activation” pathway, along with *SERPINC1, COL3A1, COL2A1* and *PROC* (Additional file 4). Only three additional targets of *JUN* (*ACAT2, FABP7* and *SLC7A*) were upregulated in liver of Rf- embryos at e15. The remaining gene targets of JUN (*DMTF1*, *Cyclin D*, *WTN9A*, *COMT, BEND4,* and *BATF3*) were expressed higher in liver of Rf + than Rf- embryos. IPA functionally annotated the second gene network (Fig. 2b) as related to “Lipid Metabolism” and “Molecular Transport”. Several genes involved in molecular transport or lipid metabolism [*APOH, APOD* (or lipocalin)*, AMBP, POYOX1, AHSG, GC, AGT, HSP90B1* and *LIPC*] were highly expressed in liver of Rf + embryos at e15. These genes are centered on high-density lipoprotein cholesterol (HDL). Additional lipolytic genes, up-regulated in the Rf + embryos, were *FKBP9, AADAC, LDAH, FAAH, PLA1A, MGLL, PROC, TBX3* and *GJA5*. Only eight genes in this network were expressed higher in the Rf- embryo liver (*PAFAH1B1, PLA2G4A, LYPLA2, PLA2R1 MIF AP1S3, FAM49A* and *ABCD2*).

**Table 4:**
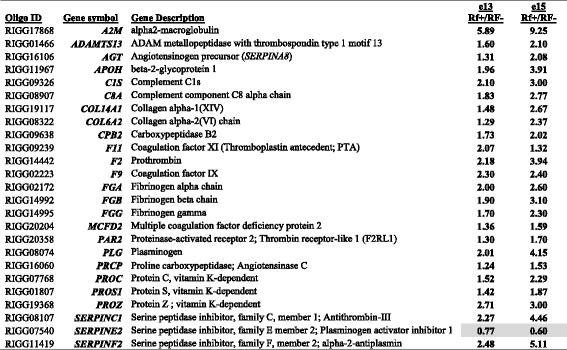
Twenty-five DE genes in liver of Rf + and Rf- embryos belonging to the blood coagulation/complement pathways

RIGG (Rosalind Institute *Gallus gallus*) oligo ID, gene symbol and description, and fold-change (Rf+/Rf- ratio) values are shown at two embryonic ages (e13 and e15). A preliminary analysis with a relaxed significance level (*P* ≤ 0.10) was completed using GeneSpring GX software (Agilent Technologies; Santa Clara, CA). Shaded expression values indicate higher expression of plasminogen activator inhibitor 1 (*PAI-1*) in liver of Rf- embryos

**Fig. 3:**
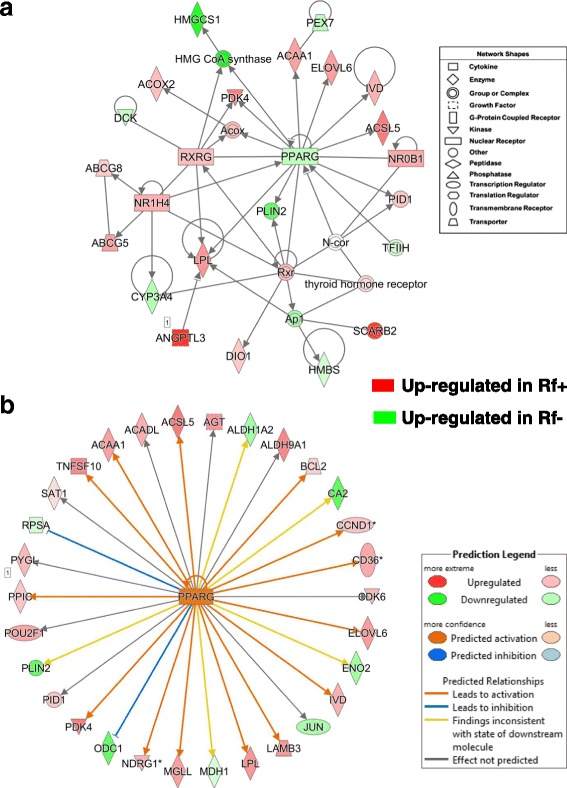
A gene network found in liver of e15 embryos showing direct interactions of ligand-activated nuclear receptors and their direct target genes controlling “Lipid Metabolism” and “Molecular Transport” (**a**). Panel **b** shows the peroxisome proliferator activated receptor gamma (*PPARG*), itself an auto-regulated gene and 30 direct target genes identified by the Ingenuity® Knowledge Base. IPA predicts, according to accrued mammalian literature, that activated PPARG would lead to either activation (orange arrows) or inhibition (blunt blue edges) of its direct (AR-DE) target genes

The network in Fig. 3a shows direct interactions among four transcription factors [peroxisome proliferator-activated receptor gamma (*PPARG*)*,* retinoid-activated receptor gamma (*RXRG*)*,* nuclear receptor subfamily 1 group H member 4 or farnesol-activated receptor (*NR1H4*) or and nuclear receptor subfamily 0 group B member 1 (*NROB1*)], which control lipid metabolism. Three of these up-stream regulators (*RXRG, NR1H4* and *NROB1*) were up-regulated in liver of Rf + embryos at e15, whereas *PPARG* was expressed high in liver of the Rf- embryos. Five additional genes (*PLIN2, CYP3A4, HMGCS1, PEX7* and *HMBS*) were higher in the Rf- embryo liver. The remaining genes, highly expressed in Rf + embryo liver, are well-known regulators of lipid metabolism [deiodinase 1 (*DIO1*), angiopoietin like 3 (*ANGPTL3*), lipoprotein lipase (LPL), acyl-CoA oxidase 2 (*ACOX2*), pyruvate dehydrogenase kinase 4 (*PDK4*), acetyl-CoA acyltransferase 1 (*ACAA1*), ELOVL fatty acid elongase 6 (*ELOVL6*), acyl-CoA synthetase long-chain family member 5 (*ACSL5*), and scavenger receptor class B member 2 (*SCARB2*)].

Ingenuity “Upstream Regulator Analysis” identified 30 AR-DE genes in Rf + and Rf- embryos at e15 that are direct target of *PPARG* (Fig. 3b) and predicts that PPARG should be activated (up-regulated) due to the large number of up-regulated genes in the e15 dataset (22 AR-DE genes). According to the Upstream Regulator Analysis, the orange-colored gene symbol for *PPARG* indicates that it should be activated, which would lead to up-regulation (orange arrows) of 22 direct target genes. However, *PPARG* was highly expressed in the liver of Rf- embryos as indicated by both microarray and qRT-PCR analyses. Eight gene targets of *PPARG* were expressed at higher levels in the Rf- liver at e15, including carbonic anhydrase 2 (*CA2*), *PLIN2*, ornithine decarboxylase 1 (*ODC1*), enolase 2 (*ENO2*), aldehyde dehydrogenase 1 family member A2 (*ALD1A2*), Jun proto-oncogene (*JUN*), malate dehydrogenase 1 (*MDH1*) and ribosomal protein SA (*RPSA*).

**Fig. 4:**
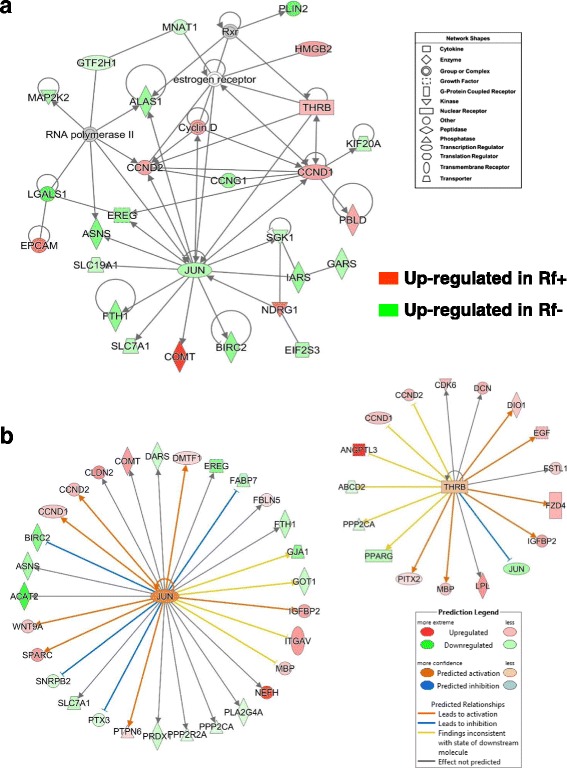
A gene interaction network involving several upstream regulators and their direct target genes involved in “Cellular Growth and Proliferation” (**a**). Ingenuity® Up-stream Regulator Analysis predicts activation of two transcription factors, JUN proto-oncogene, AP-1 transcription factor subunit (*JUN*) and thyroid hormone receptor beta (*THRB*), and their direct target genes (**b**). These gene networks were identified in a combined dataset of all AR-DE genes found in the liver at e13 and e15 (see Fig. 2b). The target genes were either upregulated in riboflavin-rescued (Rf+; red-colored symbols) or expressed higher in riboflavin-deficient (Rf-; green-colored symbols) embryo livers. IPA predicts that the two up-stream regulators are “activated”, which would lead to either activation (orange arrows) or inhibition (blunt blue edges) of their respective direct target genes

A gene network centered on the interaction of *JUN,* which is upregulated in Rf- embryos*,* with three other upstream regulators [thyroid hormone receptor beta (*THRB*), cyclin D1 and D2 (*CCND*1 and *CCND2*) is involved in “Cellular Growth and Proliferation” (Fig. 4a). This gene network was identified by IPA from a combined dataset of all AR-DE genes found in the liver at e13 and e15. Eight additional genes, expressed higher in the liver of Rf- embryos, were serum/glucocorticoid regulated kinase 1 (*SGK1*), isoleucyl-tRNA synthetase (*IARS*), baculoviral IAP repeat containing 2 (*BIRC2*; an inhibitor of apoptosis), solute carrier family 7 member 1 (*SLC7A1*), aminoacyl-tRNA hydrolase (*PTH1*), solute carrier family 19 member 1 (*SLC19A1*; a folate transporter), asparagine synthetase (glutamine-hydrolyzing) (*ASNS*) and epiregulin (*EREG*). Among the other up-regulated genes in the Rf + embryos found in this network were N-myc downstream regulated 1 (*NDRG1*), catechol-O-methyltransferase (*COMT*), epithelial cell adhesion molecule (*EPCAM*), high mobility group box 2 (*HMGB2*), and phenazine biosynthesis like protein domain containing (*PBLD*). Ingenuity Upstream Regulator Analysis identified direct targets of the transcription factors JUN and THRB (Fig. 4b). Based on the observed state of direct targets of *JUN*, Ingenuity predicts that *JUN* should be activated, leading to activation of *IGFBP2, SPARC, WNT9A, CCND1, CCND2* and *DMTF1* (orange-colored arrows). Blue-colored blunted lines predict that *JUN* would inhibit expression of *BIRC2, FABP7, PTX3* and *SNRPB2* (i.e., actually *JUN* and these target genes are expressed higher in liver of Rf- embryos). Of the 17 targets of *THRB*, the majority (13 AR-DE genes) are upregulated in liver of the Rf + embryos. Further, activation of *THRB* in the Rf + embryos would lead to downregulation of *JUN* (blunted blue line), which is the observed state of *JUN* in the e15 dataset.

**Fig. 5:**
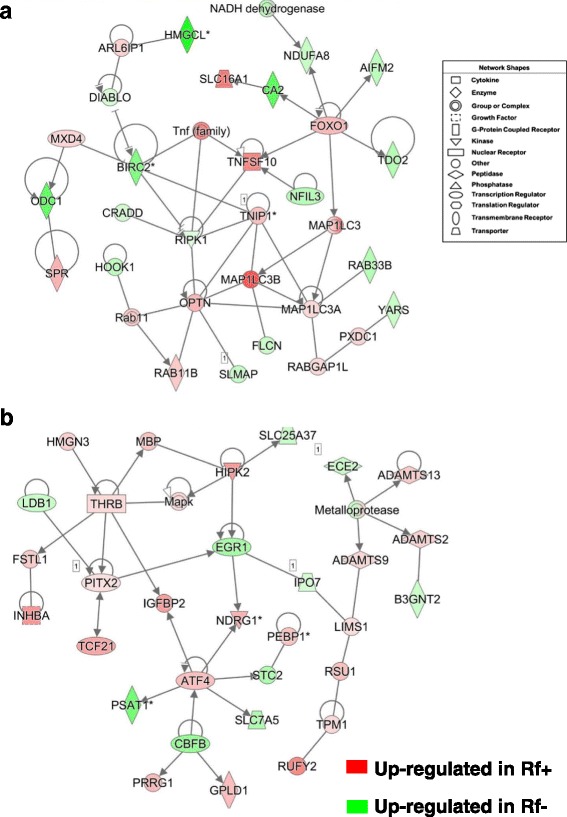
Two direct gene interaction networks regulating apoptosis, necrosis and the cell cycle. The top panel (**a**) shows a gene network functionally annotated by IPA as “Cell Death and Survival”, “Cell Morphology”**.** The bottom panel (**b**) depicts a gene network that was annotated by IPA as related to the “Cell Cycle” and “Cancer”. These interaction networks were found in the liver of riboflavin-deficient and riboflavin-rescued embryos at e15

The gene network shown in Fig. 5a was annotated by IPA as related to “Cell Death and Survival”. Eleven genes were expressed higher in Rf + embryos at e15 including the transcription factor forkhead box O1 (*FOXO1*), solute carrier family 16 member 1 (*SLC16A1*; a monocarboxylate transporter), tumor necrosis factor superfamily member 10 (*TNFSF10*), ADP ribosylation factor like GTPase 6 interacting protein 1 (*ARL6IP1*), MAX dimerization protein 4 (*MAXD4*), sepiapterin reductase (*SPR; or* 7,8-dihydrobiopterin:NADP+ oxidoreductase), RAB11B, member RAS oncogene family (*RAB11B*), optineurin (*OPTN*), microtubule associated protein 1 light chain 3 alpha and beta (*MAP1LC3A* and *MAP1LC3B*), and RAB GTPase activating protein 1 like (*RABGAP1L*). We found higher expression of several genes in liver of Rf- embryos, including tryptophan 2,3-dioxygenase (*TDO2*), the flavoprotein, apoptosis inducing factor, mitochondria associated 2 (*AIFM2*), NADH:ubiquinone oxidoreductase subunit A8 (*NDUFA8*), baculoviral IAP repeat containing 2 (*BIRC2*), 3-hydroxymethyl-3-methylglutaryl-CoA lyase (*HMGCL*), diablo IAP-binding mitochondrial protein (*DIABLO*), nuclear factor, interleukin 3 regulated (*NFIL3*), RAB33B, member RAS oncogene family (*RAB33B*), tyrosyl-tRNA synthetase (*YARS*), folliculin (*FLCN*), receptor interacting serine/threonine kinase 1 (*RIPK1*), CASP2 and RIPK1 domain containing adaptor with death domain (*CRADD*), hook microtubule tethering protein 1 (*HOOK1*) and ornithine decarboxylase 1 (*ODC1*). The network shown in Fig. 5b was functionally annotated as “Cancer” and “Cell Cycle”. Three transcription factors, thyroid hormone receptor, beta (*THRB*)*,* paired like homeodomain 2 (*PITX2*) and activating transcription factor 4 (*ATF4*) were expressed at higher levels in Rf + embryos at e15, while early growth response 1 (*EGR1*), core-binding factor beta subunit (*CBFB*) and LIM domain binding 1 (*LDB1*) were higher in liver of the Rf- embryos. Also upregulated in the Rf- embryos were solute carrier family 25 member 37 (*SLC25A37*), endothelin converting enzyme 2 (*ECE2*), importin 7 (*IPO7*), UDP-GlcNAc:betaGal beta-1,3-N-acetylglucosaminyltransferase 2 (*B3GNT2*; which is involved in hepatocarcinoma), stanniocalcin 2 (*STC2*), solute carrier family 7 member 5 (*SLC7A5*) and the anticancer gene, phosphoserine aminotransferase 1 (*PSAT1*).

**Fig. 6:**
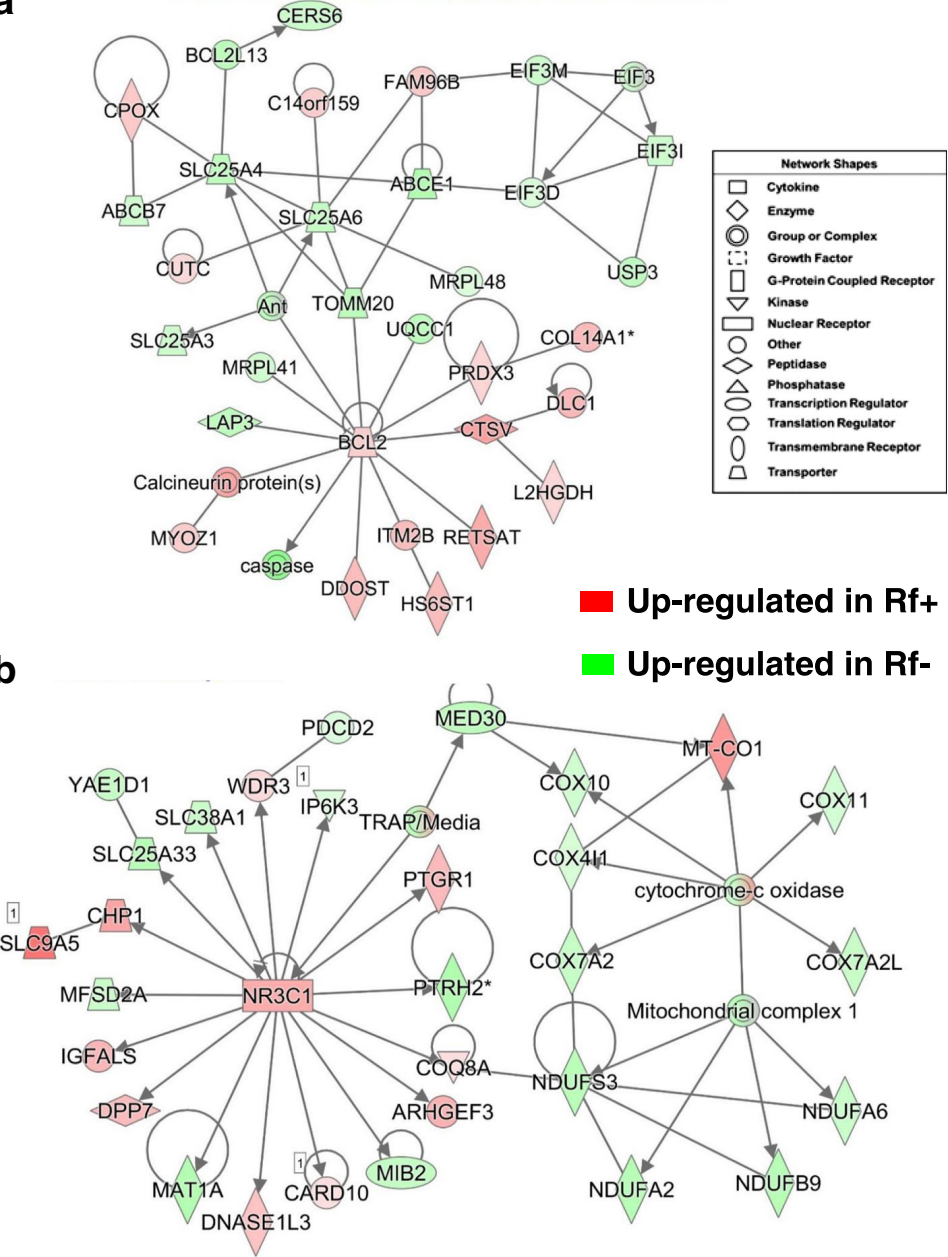
Two interaction networks found in the liver of riboflavin-deficient and riboflavin-rescued embryos at e15. The top panel (**a**) depicts a direct gene interaction network involved in “Gene Expression” and “Protein Synthesis”. This gene network illustrates direct interactions of the apoptosis regulator *BCL2* with numerous enzymes and transporters. The bottom panel (**b**) shows the glucocorticoid receptor (*GCR*) [or *NR3C1*] and several direct target genes; two of which (*MED30* and *COQ8A*) interact with subunits of mitochondrial cytochrome c oxidase (*COX*) and NADH:ubiquinone oxidoreductase (*NDUF*). This network was annotated by IPA as related to “Developmental Disorder” and “Metabolic Disease”

The gene network shown in Fig. 6a is involved in “Gene Expression and Protein Synthesis” and focused on BCL2, apoptosis regulator (*BCL2*), expressed higher in Rf + embryos at e15. Ten additional genes that interact with *BCL2* and are highly expressed in Rf + embryos, include the antioxidant peroxiredoxin 3 (*PRDX3*), collagen type XIV alpha 1 chain (*COL14A1*), cathepsin V (*CTSV*), DLC1 Rho GTPase activating protein (*DLC1*), L-2-hydroxyglutarate dehydrogenase (*L2HGDH*), retinol saturase (*RESTAT*), integral membrane protein 2B (*ITM2B*), dolichyl-diphosphooligosaccharide--protein glycosyltransferase non-catalytic subunit (*DDOST*), calcineurin (*PPP3CA*), and myozenin 1 (*MYOZ1*). *BCL2* also interacts with several genes expressed higher in the Rf- embryos [caspase (*CASP3*), leucine aminopeptidase 3 (*LAP3*), mitochondrial ribosomal protein L41 (*MRPL41*), translocase of outer mitochondrial membrane 20 (*TOMM20*), ubiquinol-cytochrome c reductase complex assembly factor 1 (*UQCC1*) and three adenine transporters (*SLCC25A3, SLC25A4* and *SLC25A6*). Additional genes found up regulated in the Rf- embryos were: ATP binding cassette subfamily B member 7 (*ABCB7*), ATP binding cassette subfamily E member 1 (*ABCE1*), BCL2 like 13 (*BCL2L13*), ceramide synthase 6 (*CERS6*), eukaryotic translation initiation factor 3, subunits D, I and M (*EIF3D, EIF3I, EIF3M*), and ubiquitin specific peptidase 3 (*USP3*). Whereas, coproporphyrinogen oxidase (*CPOX*), chromosome 14 open reading frame 159 (*C14orf159*), and family with sequence similarity 96 member B (*FAM96B*) were expressed higher in the Rf + embryos. According to IPA, the network in Fig. 6b is related to “Developmental Disorder” and “Metabolic Disease”. This network was composed of direct targets of the up-regulated glucocorticoid receptor (GCR; *NR3C1*) and their interaction with mitochondrial complexes namely, subunits of cytochrome oxidase and NADH: ubiquinone oxidoreductase, which with the exception of mitochondrial encoded cytochrome c oxidase I (*MT-CO1*) were expressed higher in liver of the Rf- embryos. Additional genes expressed higher in the Rf- embryos were *MED30*, *PTRH2*, mind-bomb E3 ubiquitin protein ligase (*MIB2*), *MAT1A, MFSD2A, YAE1D1, SLC25A33, SLC38A1, PDCD2* and *IP6K3*. The following genes were up regulated in Rf + embryos: *WDR3,* prostaglandin reductase 1 (*PTGR1), COQ8A, CARD10, DNASE1L3, DPP7, IGFALS* and *CHP1*.

**Fig. 7:**
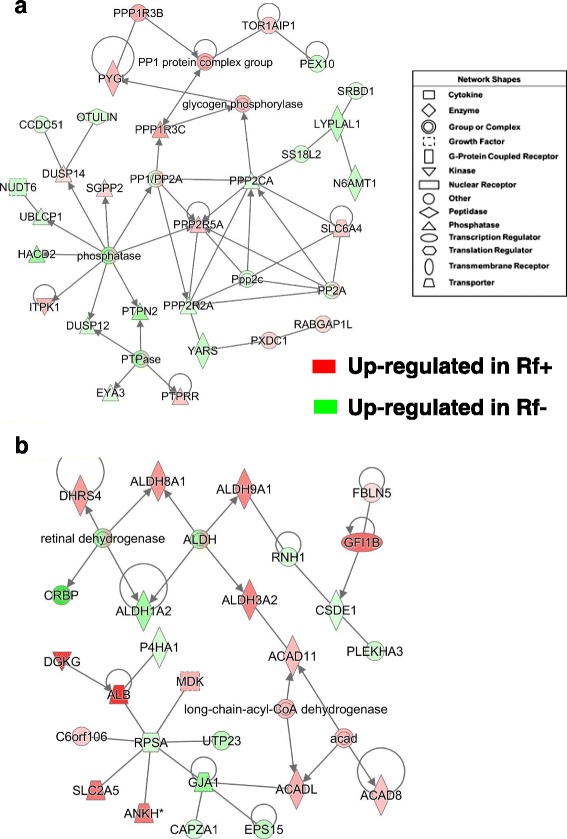
Hepatic gene interaction networks involved in phosphorylation or retinal metabolism of riboflavin-deficient and riboflavin-rescued embryos. Panel **a** shows the interaction of several phosphatases and phosphorylases, including glycogen phosphorylase (*PYGL*) and annotated by IPA as “Carbohydrate Metabolism” and “Post-Translational Modification”. This gene network was identified in the combined dataset of all AR-DE genes found at e13 and e15. The second gene network (**b**), found in liver of e15 embryos was composed mainly of acyl-CoA and aldehyde dehydrogenases, and functionally annotated by IPA as involved in “Lipid Metabolism” and “Small Molecule Biochemistry”

The network shown in Fig. 7a is composed of several phosphatases that are involved in “Carbohydrate Metabolism” and “Post-Translational Modification”. This gene network was identified in a combined dataset of all AR-DE genes found in the liver at e13 and e15. Several phosphorylases, including glycogen phosphorylase L (*PYGL*), protein phosphatase 1 regulatory subunit 3B (*PPP1R3B*) and 3C (*PPP1R3C*), protein phosphatase 2 regulatory subunit B-alpha (*PPP2R5A*), protein tyrosine phosphatase, receptor type R (*PTPRR*), inositol-tetrakisphosphate 1-kinase (*ITPK1*), and dual specificity phosphatase 14 (*DUSP14*), and sphingosine-1-phosphate phosphatase 2 (*SGPP2*) were expressed higher in liver of Rf + embryos. Other up-regulated genes in the Rf + embryos include PX domain containing 1 (*PXDC1*), torsin 1A interacting protein 1 (*TOR1AIP1*), RAB GTPase activating protein 1 like (*RABGAP1L*), and the serotonin transporter (*SLC6A4*). Sixteen additional genes in this network are expressed higher in Rf- embryos including (*PEX10*), S1 RNA binding domain 1 (*SRBD1*), lysophospholipase like 1 (*LYPLAL1*), SS18 like 2 (*SS18L2*), N-6 adenine-specific DNA methyltransferase 1 (*N6AMT1*), protein phosphatase 2 catalytic subunit alpha (*PPP2CA*), protein phosphatase 2 regulatory subunit B’alpha (*PPP2R2A*), tyrosyl-tRNA synthetase (*YARS*), EYA transcriptional coactivator and phosphatase 3 (*EYA3*), protein tyrosine phosphatase, non-receptor type 2 (*PTPN2*), dual specificity phosphatase 12 (*DUSP12*), 3-hydroxyacyl-CoA dehydratase 2 (*HACD2*), ubiquitin like domain containing CTD phosphatase 1 (*UBLCP1*), nudix hydrolase 6 (*NUDT6*), coiled-coil domain containing 51 (*CCDC51*) and OTU deubiquitinase with linear linkage specificity (*OTULIN*).

The gene network in Fig. 7b is composed of several aldehyde dehydrogenases (*ALDH8A1, ALDH9A, ALDH3A2* and *ALDH1A2*) and acyl-CoA dehydrogenases (*ACAD11, ACADL* and *ACAD8*); according to IPA, these genes are involved in “Lipid Metabolism and Small Molecule Biochemistry”. The short-chain dehydrogenase/reductase 4 (*DHRS4*) was expressed higher in the Rf + embryos, whereas the retinol binding protein 1 (*RBP1* or *CRBP*) and *ALDH1A2,* which catalyzes the synthesis of retinoic acid (RA), were more abundant in liver of the Rf- embryo on e15. Additional up-regulated genes found in the Rf + embryo include fibulin 5 (*FBLN5*), growth factor independent 1B transcriptional repressor (*GFI1B*), ANKH inorganic pyrophosphate transport regulator (*ANKH*), fructose transporter (*SLC2A5*), albumen (*ALB*), diacylglycerol kinase gamma (*DGKG*) and midkine (neurite growth-promoting factor 2; *MDK*). Nine additional genes in this network (*RNH1, CSDE1, PLEKHA3, EPS15, CAPZA1, GJA1, RPSA, UTP23* and *P4HA1*) were expressed higher in liver of Rf- embryos at e15.

**Fig. 8:**
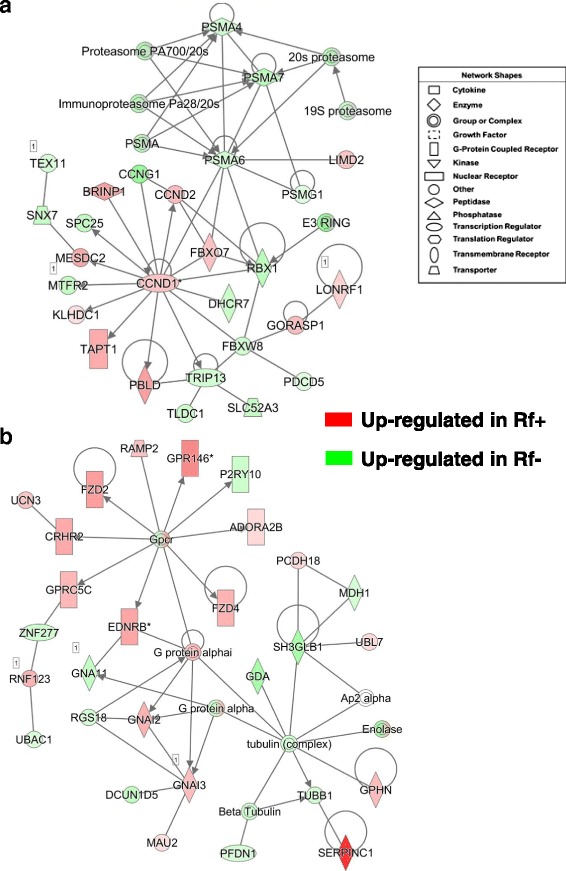
Gene interaction networks controlling the proteasome (**a**) and cell signaling via G-protein coupled receptors (**b**) in liver of riboflavin-deficient and riboflavin-rescued embryos at e15. Panel **a** shows interactions between components of the proteasome and direct target genes of cyclin D (*CCND1*) and annotated by IPA as “Neurological Disease”. The gene network in Panel **b** depicts interactions of several G-protein coupled receptors (GPCRs) and members of the tublin complex. This network was functionally annotated by IPA as related to “Hematological Disease” and “Cell Signaling”

The gene network in Fig. 8a shows the interaction of proteasome subunits (*PSMA4, PSMA6, PSMA7* and *PSMG1*) with the transcription factor cyclin D1 (*CCND1*) and its direct targets in liver of e15 embryos. The expression of *CCND1*, F-box protein 7 (*FBXO7*), *CCND2*, BMP/retinoic acid inducible neural specific 1 (*BRINP1*), mesoderm development candidate 2 (*MESDC2*), transmembrane anterior posterior transformation 1 (*TAPT1*) and phenazine biosynthesis like protein domain containing (*PBLD)* were up regulated in liver of Rf + embryos. Among genes that were expressed higher in the Rf- liver and directly related to *CCND1* were ring-box 1 (*RBX1*), 7-dehydrocholesterol reductase (*DHCR7*), F-box and WD repeat domain containing 8 (*FBXW8*), thyroid hormone receptor interactor 13 (*TRIP13*), mitochondrial fission regulator 2 (*MTFR2*), kinetochore complex component (*SPC25*), and *CCNG1*. Additional genes expressed higher in the Rf- embryo were the riboflavin transport protein solute carrier family 52 member 3 (*SLC52A3*), TBC/LysM-associated domain containing 1 (*TLDC1*), programmed cell death 5 (*PDCD5*), sorting nexin 7 (*SNX7*), and testis expressed 11 (*TEX11*). IPA functionally annotated the gene network in Fig. 8b as related to “Hematological Disease and Cell Signaling”. This network is composed of a cluster of G-protein coupled receptors (GCPRs), including adenosine A2b receptor (*ADORA2B*), purinergic receptor P2Y10 (*P2RY10*), G protein-coupled receptor 146 (*GPR146*), frizzled class receptor 2 (*FZD2*), *FZD4*, corticotropin releasing hormone receptor 2 (*CRHR2*), G protein-coupled receptor class C group 5 member C (*GPRC5C*), endothelin receptor type B (*EDNRB*), and guanine nucleotide binding protein (G protein), alpha inhibiting-2 and -3 (*GNAI2* and *GNAI3*). All of these genes, except *P2RY10*, were expressed higher in Rf + embryos. The tubulin cluster [tubulin beta 1 (*TUBB1*)] interacts G protein alpha, prefoldin subunit 1 (*PFDN1*), guanine deaminase (*GDA*), malate dehydrogenase 1 (*MDH1*), via H3 domain containing GRB2 like, endophilin B1 (*SH3GLB1*), and enolase (*ENO*), which are all expressed at higher abundance in Rf- embryos at e15. Interestingly, serpin family C member 1 (*SERPINC1* or anti-thrombin 3), gephyrin (*GPHN*], protocadherin 18 (*PCDH18*) and ubiquitin like 7 (*UBL7*) were up-regulated DE genes in the Rf + embryos.

### Verification of differential gene expression in Rf + and Rf- embryos by qRT-PCR analysis

**Fig. 9:**
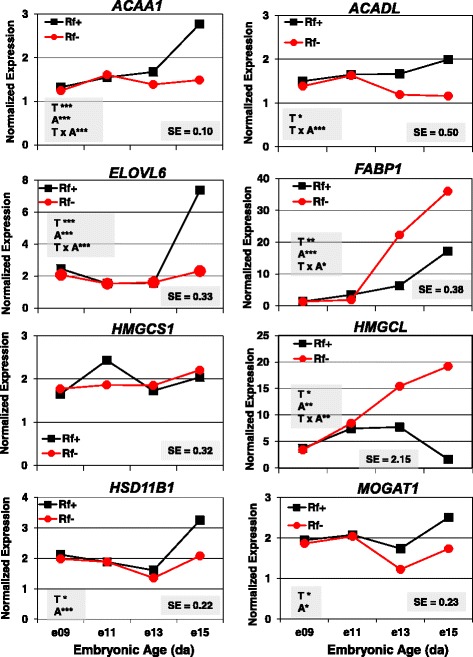
Verification of differential expression of eight metabolic genes in liver of riboflavin-deficient chick embryos by qRT-PCR analysis. The abundance of eight metabolic genes was determined in 4 biological samples/treatment (Rf + or Rf-) at 4 embryonic ages (e9, e11, e13 and e15). Data were analyzed by ANOVA using the GLM procedure in SAS and mean separation using a protected Fisher’s least significant difference (LSD) test. The shaded insets show the common standard error (SE) of the least squares mean (LSMEAN) and significant differences (* *P* ≤ 0.05, ** *P* ≤ 0.01 or *** *P* ≤ 0.001) for the main effects of treatment (T) and age (A) or their interaction (T x A)

Twenty-four “candidate” genes were initially selected based on biological function for verification of differential expression by qRT-PCR analysis across four embryonic ages (e9, e11, e13 and e15), along with 6 invarant housekeeping genes (see Additional file 2). The qRT-PCR analysis was performed on the same 32 RNA samples that were used for the microarray analysis. As found in microarray analysis, qRT-PCR analysis showed no significant differences in expression of eight metabolic genes between the Rf + and Rf- embryos at e9 or e11 (Fig. 9). The expression of *ACAA1, ACADL, ELOVL6, HSD11B1* and monoacylglycerol O-acyltransferase 1 (*MOGAT1*) was higher in liver of Rf + embryos at e15. On the other hand, the abundance of the hepatic *FABP1* and the ketogenic enzyme *HMGCL* was greater in liver of the Rf- embryos at e13 and e15.

**Fig. 10:**
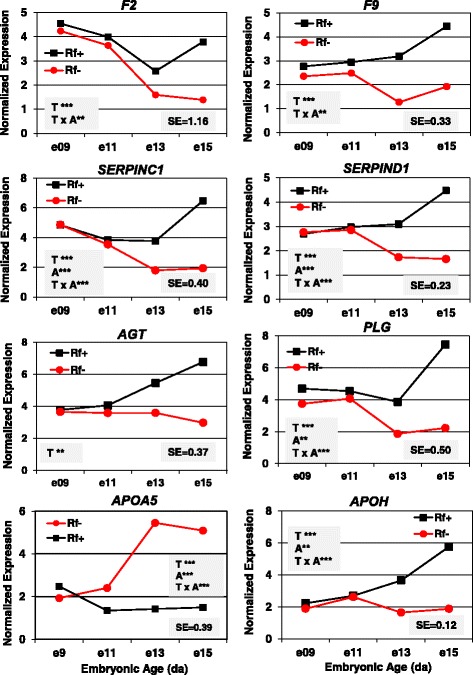
Verification of differential gene expression in riboflavin deficient chick embryos by qRT-PCR analysis. The hepatic abundance of six coagulation factors (*F2, F9, SERPINC1, SERPIND1, AGT* and *PLG*) and two lipid transporters (*APOA5* and *APOH*) was determined in 4 biological samples/treatment (Rf + or Rf-) at 4 embryonic ages (e9, e11, e13 and e15). Data were analyzed by ANOVA using the GLM procedure in SAS and mean separation using Fisher’s protected least significant difference (LSD) test. The shaded insets show the common standard error (SE) of the least squares mean (LSMEAN) and significant differences (* *P* ≤ 0.05, ** *P* ≤ 0.01 or *** *P* ≤ 0.001) for the main effects of treatment (T) and age (A) or their interaction (T x A)

The greatest differences in expression of six hemostasis genes (Fig. 10) were found between Rf + and Rf- embryos at e15, where Rf- embryos had a lower abundance as indicated by a significant (*P* ≤ 0.01-*P* ≤ 0.001) main effect of riboflavin treatment (T) or a treatment by age (T x A) interaction (*F2, F9, SERPINC1, SERPIND1* and *PLG*). The expression of apolipoprotein A5 (*APOA5*), which controls plasma triglyceride levels, was 3-fold higher in the liver of Rf- embryos at e13 and e15. The *APOH* transcript was more abundant in liver of Rf + embryos at e13 and e15, as indicated by a highly significant (*P* ≤ 0.001) treatment x age interaction.

**Fig. 11:**
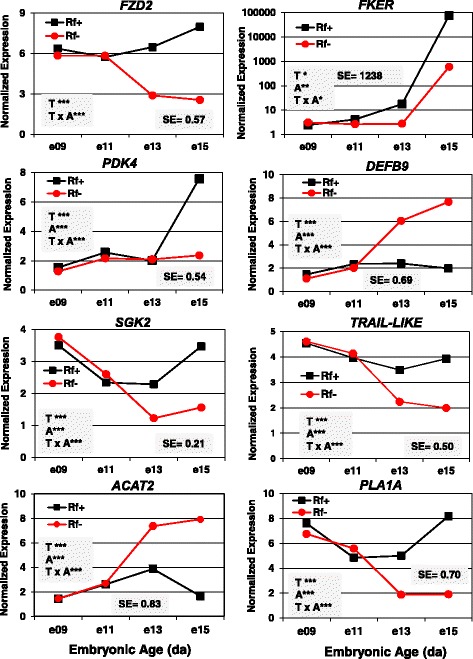
Verification of differential expression of eight DE genes in riboflavin deficient and rescued embryos by qRT-PCR analysis. The hepatic expression of eight DE genes was verified by independent qRT-PCR analysis of the same RNA sample used in microarray analysis. The eight genes include frizzled class receptor 2 (*FZD2*), feather keratin (*FKER*), defensin beta 9 (*DEFB9*), kinases (*PDK4, SGK2*), a phosphatase (*PSPH*), acetyl-CoA acetyltransferase 2 (*ACAT2*), and phospholipase A1 member A (*PLA1A*). Data were analyzed by ANOVA using the GLM procedure in SAS and mean separation using Fisher’s protected least significant difference (LSD) test. The shaded insets show the common standard error (SE) of least squares mean (LSMEAN) and significant differences (* *P* ≤ 0.05, ** *P* ≤ 0.01 or *** *P* ≤ 0.001) for the main effects of treatment (T) and age (A) or their interaction (T x A)

The expression of six hepatic genes [frizzled homolog 2 (*FZD2*), feather keratin (*FKER*), pyruvate dehydrogenase kinase, isozyme 4 (*PDK4*), serum/glucocorticoid regulated kinase 2 (*SGK2*), TNF-related apoptosis inducing ligand-like (*TRAIL-LIKE*) and phospholipase A1 member A (*PLA1A*) was higher in Rf + embryos at e13 and e15 (Fig. 11). Remarkably, the abundance of feather keratin (*FKER*) was 100-times greater in liver of Rf + embryos at e15 than that found in Rf- embryos. In contrast, the expression of the antimicrobial peptide β-defensin 9 (*DEFB9*) and acetyl-CoA acetyltransferase 2 (*ACAT2*) were 2-fold to 3-fold greater in liver of the Rf- embryos between e13 and e15.

**Fig. 12:**
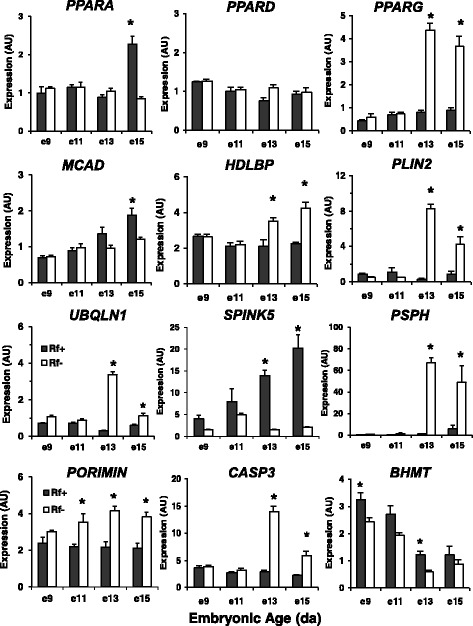
The hepatic expression of 12 additional “candidate” genes was examined by qRT-PCR analysis in riboflavin-deficient and riboflavin-rescued embryos. The abundance of 12 additional genes was determined in an identical set of 32 RNA samples representing 4 biological samples/treatment (Rf + or Rf-) at 4 embryonic ages (e9, e11, e13 and e15). These gene expression levels are presented as arbitrary units (AU) and represent the mean ± SEM of four embryos/treatment and age. The Student’s *t*-test was used at each age to determine significant differences (*P* ≤ 0.05) between Rf + and Rf- embryos

An additional qRT-PCR analysis was completed on 12 genes of special interest (Fig. 12). In this case, significant differences (*P* < 0.05) between riboflavin treatments at each age were determined by the Student’s T-test. The expression of peroxisome proliferator activated receptor alpha (*PPARA*) was higher in the Rf + embryos at e15, while *PPARG* levels were sharply elevated in liver of the Rf- embryos at e13 and e15. In contrast, the hepatic expression of *PPARD* was not affected by riboflavin availability or embryonic age. The abundance of medium chain acyl-CoA dehydrogenase (*MCAD*) was lower in Rf- embryos at e15. The expression of high-density lipoprotein binding protein (*HDLBP*), perilipin 2 (*PLIN2*) [or adipose differentiation-related protein (*ADRP*)], ubiquilin 1 (*UBQLN1*) and the apoptosis inducer caspase 3 (*CASP3*) were also higher in the Rf- embryos at e13 and e15. Porimin (*PORIMIN*) transcript levels were significantly higher in the Rf- embryos at e11, e13 and e15. Porimin is a unique cell surface receptor that mediates oncotic cell death, characterized by rupture of the cell membrane without DNA fragmentation. Serine peptidase inhibitor, Kazal type 5 (*SPINK5*) [or ovoinhibitor (*OIH*)] expression increased progressively in liver of the Rf + embryo and reached highest levels (*P* ≤ 0.05) at e13 and e15. The pattern of phosphoserine phosphatase (*PSPH*) expression was similar to that of *PLIN2*, being sharply up-regulated in liver of Rf- embryos between e13 and e15. Betaine-homocysteine S-methyltransferase (*BHMT*) transcript abundance was consistently lower in liver of Rf- embryos, reaching significance at e9 and e13.

For verification of differential gene expression, the Pearson’s correlation coefficient [r] was determined for 21 “candidate” genes using normalized expression values (log2 ratio of Rf+/Rf-) averaged across two ages (e13 and e15) from the qRT-PCR analysis versus the 20.7 K oligo microarray analysis (Additional file 6). The calculated Pearson’s coefficient (*r* = 0.834) from this data set indicates a highly significant (*P* ≤ 0.01) association between normalized expression values determined by microarray analysis and qRT-PCR analysis, as we have shown previously [30, 31].

## Discussion

Previously, we have shown diminished activity of several flavin-dependent enzymes in riboflavin-deficient chicken embryos, disruption of fatty acid oxidation, alternative energy production, hypoglycemia, and sudden death in mid-embryonation [9, 10, 14, 16, 35, 36]. In the present paper, we describe time-course (e9-e15) transcriptional responses of liver in riboflavin-deficient and riboflavin-rescued embryos. The general disruption of β-oxidation of fatty acid causes excessive accumulation of lipid in liver of riboflavin-deficient embryos (see Fig. 1a), as reflected by higher expression of genes involved in adipogenesis and intracellular lipid storage (*PPARG, APOA5, PLIN2, DEFB9* and *CYP51A1*). Blood coagulation factors were among the most highly-expressed genes found in liver of riboflavin-rescued embryos, particularly genes belonging to the “Intrinsic Prothrombin Activation” cascade. A more extensive and inclusive cluster of 25 hemostasis/clotting genes (see Table 4) was revealed by our initial analysis of all 32 microarrays representing four riboflavin-deficient and four riboflavin-rescued embryos across four embryonic ages (e9-e15). Twenty-four genes belonging to the blood coagulation/complement pathway were expressed at higher abundance in liver of riboflavin-rescued embryos at e13 and e15. The only exception was over-expression of a single gene *SERPINE2* (or plasminogen activator inhibitor 1, *PAI-1*) in liver of the riboflavin-deficient embryos at e13 and e15. Plasminogen activator inhibitor 1 belongs to the fibrinolytic pathway which dissolves the fibrin embedded in the blood clot. This suggests that increased hepatic expression of *PAI-1* in riboflavin-deficient embryos represents an attempt to prevent blood loss, while faced with a failed coagulation system at multiple levels. In riboflavin-deficient embryos, higher expression of hepatic genes that enhance protein breakdown via activation of the ubiquitin-proteome pathway eventually leads to apoptosis and programmed cell death. Our present transcriptional study of riboflavin-deficient and riboflavin-rescued embryos clearly shows the importance of a single required vitamin—riboflavin—across multiple critical biological processes (β-oxidation of lipid, euglycemia, blood coagulation and feathering).

### Riboflavin deficiency and impaired lipid metabolism

The most obvious difference in metabolism between Rf-deficient and Rf-rescued embryos was the excessive accumulation of lipid (see Fig. 1a) and intermediates of fatty acid oxidation in liver of the Rf- embryos [16]. The natural surge in lipid transfer from yolk to the liver in mid-embryonic development [6, 37] is reflected by a metabolic shift toward exclusive oxidation of lipid as indicated by a respiratory quotient of 0.72 [15] until the chick hatches and consumes its first meal. Among biological pathways that had notable differences in differential expression in genes were those of lipid metabolism, where two-thirds of the 66 AR-DE genes were expressed higher in the Rf + embryos (Additional file 5). Fatty acid binding protein 1 (*FABP1*) and fatty acid binding protein 7 (*FABP7*) were expressed higher in liver of the Rf- embryos at e15. These genes belong to a family of cytoplasmic hydrophobic ligand-binding proteins, which are involved in binding and intracellular transport of long-chain fatty acids. In the heart and brain, FABP7 is thought to be involved in regulating the supply of fatty acids for mitochondrial β-oxidation [38]. Perilipin (*PLIN2*) is found in the vascularized yolk sac of the chick embryo [39] and associated with accumulation of lipid droplets in cells, particularly adipocytes. A number of genes encoding enzymes involved in recycling Coenzyme A (CoA) were more abundant in liver of Rf + embryos, including acyl-CoA synthase medium-chain 3 (*ACSM3*), acyl-CoA dehydrogenase, long chain (*ACADL*) and acetyl-CoA acyltransferase 1 (*ACAA1*). In contrast, the liver of Rf- embryos have higher expression of acetyl-CoA acetyltransferase 2 (*ACAT2*) and genes that drive ketogenesis [3-hydroxymethyl-3-methylglutaryl-CoA lyase (*HMGCL*) and 3-hydroxy-3-methylglutaryl-CoA synthase 1 (*HMGCS1*)], which were among the most highly expressed genes found in the Rf- embryo at e15. Thus, riboflavin deficiency in chicken embryos has a great impact on β-oxidation, since it involves three successive flavin-dependent enzymes [14]. We found a depression in medium chain acyl-CoA dehydrogenase (*MCAD*) transcripts in the riboflavin-deficient chick embryos. In humans, MCAD deficiency (MCDD) leads to sudden death of breast-feeding infants, especially following a prolonged overnight fast [18]. Although Rf- embryos also experience sudden death [14], the etiology in *rd/rd* embryos reflects the global impact of flavin deficiency, rather that deficiency of a single flavin-dependent enzyme [i.e., MCAD or acyl-CoA dehydrogenase (ACADM)]. Furthermore, the constraints of embryonic development within the cleidoic egg of amniotes require a specialized transport system, the riboflavin binding protein (RBP), to transport essential riboflavin from the dam’s liver to the oviduct for deposition into the fertilized egg. However, the *rd/rd* genotype does not affect the intestinal riboflavin transport system, since both parents and hatchling chicks with this genotype receive an adequate supply of essential riboflavin (Vitamin B2) from their respective diets for normal growth and development.

Two ligand-activated nuclear receptor pathways were overpopulated by AR-DE genes in liver of Rf + embryos [“LXR-RXR (19/1) and FXR-RXR (17/1) Activation Pathways”]. The LXR is the major regulator of lipogenesis in chicken liver, controlling lipogenic transcription factors and lipogenic enzymes [40]. Farnesoid X receptor (FXR) is a ligand-activated nuclear receptor that serves as a receptor for bile acids and a heterologous partner with RXR. Mutations in the FXR gene lead to familial neonatal cholestasis, a serious metabolic condition caused by reduced bile secretion and fat absorption from the gut [41].

And despite the availability of yolk lipids and oxygen, mitochondrial oxidative metabolism in Rf- embryos shuts down. The impairment of fatty acid β-oxidation results in the accumulation of fatty acyl Coenzyme A (CoA) intermediates which in turn depletes the pool of free CoA needed to generate acetyl CoA, the point of entry into the citric acid cycle and production of ATP via the electron transport system and oxidative phosphorylation. Furthermore, other oxidative pathways activated by flavin-dependent enzymes and CoA would be similarly crippled (i.e., certain aliphatic amino acids and ketogenic pathways). In fact, both enzymes that drive ketogenesis (*HMGCL* and *HMGCS1*) were among the highest expressed genes found in liver of Rf- embryos at e13 and e15 (see Table 3; Figs. 3a and 9). The enzyme, 3-hydroxymethyl-3-methylglutaryl-CoA lyase (*HMGCL*), catalyzes cleavage of HMG-CoA into acetyl-CoA and acetoacetate; a key step in ketogenesis, which provides the energy required by non-hepatic tissues, particularly during fasting or starvation [42]. In the present case, enhanced expression of ketogenic genes in liver of riboflavin-deficient chick embryos could be an attempt to implement this pathway; which is blocked by their inability to oxidize yolk lipids amassed in liver. With diminution of mitochondrial ATP, non-oxidative glycolytic metabolism generates ATP until glycogen reserves are depleted. A last resort response to prevent metabolic failure and hypoglycemia in Rf- embryos seems to be increased protein catabolism, as indicated by an activated proteome and enhanced catabolism of amino acids in liver of Rf- embryos (Tables 2 and 3; Figs. 8a and 12).

Impaired lipid metabolism was evident in the distribution of the 66 AR-DE genes involved in Fatty Acid Metabolism; 45 genes were expressed higher in the Rf + embryos, whereas only 21 AR-DE genes were more abundant in the Rf- embryos (see Additional file 5). The number of AR-DE genes in several other metabolic processes (Oxidation of Lipid, Carbohydrate Metabolism, Cleavage of Carbohydrates, and Catabolism of Protein) were also 2-fold greater in the Rf + embryos than Rf- embryos. Nineteen AR-DE genes in the e15 embryos were identified as flavoproteins and of these, 15 AR-DE genes were expressed higher in Rf + liver than Rf- embryos. The large number of up-regulated genes in Rf + embryos encoding flavoproteins demonstrates a positive response to riboflavin rescue since their gene expression ratio was greater, which we consider as normal.

The dependence of chicken embryos upon riboflavin seems to be more extensive in late development (e13 and e15), when lipid catabolism becomes critical for rapid growth and development of the embryo. Impaired lipid metabolism (despite lipid-engorged livers), massive hemorrhaging, delayed feathering, apoptosis and sudden death [14] found in riboflavin-deficient embryos clearly demonstrates the importance of this single nutrient—riboflavin—across multiple biological processes during mid-embryonic development and the chick embryo’s absolute dependence upon yolk-lipid as the final fuel required for growth and hatching. This critical step is unavailable to *rd/rd* embryos due to a mutation in the hen’s gene encoding riboflavin-binding protein (*RBP*) which prevents riboflavin transport into the fertilized egg. This metabolic catastrophe can be avoided by a single injection of a trace amount of riboflavin into the *rd/rd* fertilized egg prior to onset of incubation. The consistent pattern across multiple pathways suggests a common riboflavin-dependent mechanism; although, neither the exact mode of action nor the biological pathways that depend upon riboflavin still remain obscure.

### Riboflavin deficiency and impaired blood clotting

We found uniform depression in expression of multiple coagulation factors (i.e., proteases, protease inhibitors and vitamin-dependent cofactors) in liver of Rf- embryos. Furthermore, riboflavin-deficient embryos present visually with massive hemorrhage in skin and visceral organs (see Fig. 1a). This malady in blood coagulation of Rf- embryos seems related to general inhibition of both *pro-coagulation* factors [*F2, F9, F11*, *FGA, FGB*, and *FGG*; thrombin-activated fibrinolysis inhibitors (carboxypeptidase B2 (*CPB2*)] and *anti-coagulation* factors [antithrombin3 (*AT3*) or *SERPINC1*), plasminogen (*PLG*), vitamin K-dependent inhibitors (*PROC, PROS1,* and *PROZ*), and alpha-2-antiplasmin (*SERPINCF2*)]; all of which were expressed at higher levels in liver of Rf + embryos. A notable exception was the over-expression of plasminogen activator inhibitor 1 (*PAI-1*) in liver of Rf-deficient embryos at e13 and e15 (see Table 4), which would lead to inhibition of fibrinolysis. High levels of endogenous PAI-1 are known to cause thrombophilia in humans due to marked inhibition of fibrinolysis [43], while over-expression of PAI-1 in liver of the Rf- embryo could represent an attempt to promote blood clotting in riboflavin-deficient embryos already experiencing marked hemophilia. It is tempting to speculate that the broad effect of riboflavin deficiency across both pro-coagulation and anti-coagulation pathways could be due to a general lack of phospholipids, since phospholipids are required cofactors for activation of the blood coagulation cascade [44]. This idea is supported by our observation of impaired lipid metabolism and phosphorylation in liver of the riboflavin-deficient embryos.

While riboflavin appears to promote expression of genes involved in acute phase response signaling and the coagulation cascade in the liver of *rd/rd* chick embryos, exposure of chick embryos to long-chain perfluoroalkyl compounds depresses transcription of numerous acute-phase response and coagulation genes in liver of e19 embryos [45], which demonstrates that these pathways are functional and responsive in mid-to-late chick embryos. The energy for this period of embryonic development exclusively depends upon the ability to utilize the lipid stored in liver and β-oxidation of lipid depends upon flavin-dependent enzymes. Our present transcriptional analysis of liver in *rd/rd* chick embryos has identified 15 flavin-dependent enzymes that respond positively to riboflavin rescue (see Additional file 5). The human flavoproteome is composed of 90 flavin-dependent enzymes, which catalyze oxidation-reduction reactions in major metabolic pathways (i.e., the citric acid cycle, β-oxidation and degradation of amino acids) [46]. Our current analysis of the flavoproteome in the chicken genome (build *Galgal*5) indicates a similar number of flavin-dependent homologs in the chicken. The present microarray analysis clearly demonstrates an extensive dependence of the hemostasis system in late cleidoic embryos upon an essential nutrient—riboflavin. The massive cutaneous and visceral hemorrhaging observed in *rd/rd* chicken embryos (see Fig. 1a) appears to be a direct consequence of riboflavin deficiency. The riboflavin-deficient embryos are obviously lacking the normal ability to control excessive cutaneous and visceral bleeding. This observation argues that riboflavin is required for normal acquisition of a functional coagulation system in chick embryos, just as Vitamin K is required for hepatic synthesis of several coagulation precursors secreted into the bloodstream.

### Riboflavin deficiency and impaired cutaneous feathering

The possibility of riboflavin deficiency causing abnormal feathering of chicken embryos was addressed by several earlier studies [47, 48], although these approaches and mixing of genetic lines limit direct comparison with our transcriptional study on riboflavin-deficient (*rd/rd*) embryos derived from Single-comb White Leghorn (SCWL) hens carrying the *rd/rd* gene. Therefore, our discovery of depressed expression of the feather keratin (*F-KER*) gene in liver of riboflavin-deficient embryos is of special interest, especially since the Rf- embryos at e15 exhibit impaired feathering with only sparse clubbed down (see Fig. 1a). This physical evidence of abnormal feathering in Rf- embryos is supported by our observation of three differentially-expressed oligo spots (RIGG10897, RIGG14163 and RIGG14953) annotated as *FKER,* which showed a 19-fold increase in abundance in the liver of Rf + embryo between e13 and e15. An even greater log-fold difference in hepatic *FKER* expression between Rf + and Rf- embryos was verified by qRT-PCR analysis (see Fig. 10). Furthermore, we originally identified *FKER* as a DE gene in liver of Rf + and Rf- embryos in an identical preliminary study using our 14 K Del-Mar Chicken Integrated Systems microarray [49], where the differentially expressed cDNA clone (pgf1n.pk001.j5 or BI064513) was sequenced from a normalized abdominal fat cDNA library [50]. This expressed sequence tag (pgf1n.pk001.j5) for *FKER* (or *FK1*) was mapped to chicken chromosome 1 (*GGA1*), which represents one of several loci for *FKER* (*GGA2, GGA5, GGA25* and *GGA27*) in the chicken genome [51]. Furthermore, phylogenetic duplication and expansion of feather diversity have expanded this multi-gene family to 111 complete β-keratin gene sequences across six chromosomes (*GGA1, GGA2, GGA5, GGA6, GGA25*, and *GGA27*) in the chicken [51]. To our knowledge, the present transcriptional study represents the first report of *FKER* (*FK1*) expression in the liver of chicken embryos. However, the relationship between hepatic expression of *FKER* and normal expression of *FKER* and other β-keratins in skin of the chicken remains unknown. Our EST clone (BI064513) derived from a normalized abdominal fat cDNA library and the present hepatic expression of *FKER* indicate that the *FKER* gene is also expressed in tissues other than skin of the chicken. A tangible explanation of the highly-expressed *FKER* transcripts found presently in liver of riboflavin-rescued chick embryos at e15 is provided by a recent transcriptional study of the “lactating” crop sac in broody pigeons [52]. Apparently, beta keratins, including feather keratins, play an important structural role in the pigeon’s crop and its production of lipid-laden crop milk, which is fed to altricial squabs at frequent intervals. Thus, the increase in expression of *FKER* in liver of e15 chick embryos could be an adaptive response that deals with the abrupt influx of yolk lipids needed for the embryo-to-hatchling transition. However, elucidation of the relationship between depressed expression of *FKER* in liver of Rf- embryos and cutaneous feathering, or other impaired biological processes (i.e., blood coagulation) will require further exploration.

The timing of feather formation [53–56] coincides with the metabolic crisis of excessive lipid accumulation in liver of riboflavin-deficient embryos and their inability to utilize the stored lipid in liver. It is possible that keratin synthesis was impaired by the lack of ATP or amino acids (i.e., l-glycine, l-proline and l-serine) available for protein synthesis at a time when amino acids generated from protein catabolism are forced into oxidative pathways for energy production. Feather-keratin is involved in the elongation of barbule cells in feathers where small proteins (i.e., β-keratins) rich in glycine, serine, and proline have evolved in birds to reinforce the mechanical resistance of feathers [57]. Interestingly, retinoic acid signaling appears to be important for the timing and expression of FKR and other β-keratins in the avian epidermis [58]. In the present study, we found differential expression of several enzymes involved in retinol metabolism (see Additional file 5), retinol transport and over-representation of RXR signaling in liver of *rd*/*rd* chick embryos at e15 (see Table 3).

One of the most highly-expressed genes found in the liver of riboflavin-deficient embryos was phosphoserine phosphatase (*PSPH*), whose abundance was more than 20-times greater in liver of the Rf- embryos at e13 and e15 (see Fig. 12). This enzyme is associated with the last step in serine biosynthesis and the release of phosphate from phosvitin, a vitellogenin protein fragment composed of more than 100 phosphoserine residues, which represents about 80% of the total phosphorus in the hen’s egg yolk [59]. Thus, phosvitin is an important source of phosphate for normal development of the avian embryo [36]. This large increase in *PSPH* found in liver of riboflavin-deficient embryos could reflect the riboflavin-deficient embryos’ increased biosynthesis of *l*-serine. However, cutaneous feathering, beak formation and scale/claw development on appendages are greatly impaired in the riboflavin-deficient embryos, despite the possible abundance of *l*-serine—a major component of β-keratin in feathers [57].

### Riboflavin deficiency enhances protein catabolism, translation initiation and programed cell death

The Rf- embryos showed higher expression of 18 hepatic genes involved in protein ubiquitination, mainly those encoding proteasome subunits (see Additional file 5) and several members of the DnaJ heat shock protein family, which stimulate ATPase activity. Eukaryotic Initiation Factor 2 (E1F2) signaling was also enriched in the liver of riboflavin-deficient embryos as indicated by higher expression of 26 genes encoding a mixture of EIF subunits and ribosomal proteins (see Additional file 4). Up-regulated apoptosis genes found in the liver of Rf- embryos at e15 include *BIRC2*, *CASP3, DIABLO, JUN* and *MAP2K2*. The initiator caspase *CASP3* was more abundant in liver of Rf- embryos at e13 and e15 which indicates activation of programmed cell death (apoptosis). Three dynamic processes are involved in apoptosis: the cleavage of effector caspases by initiator caspases, the cytosolic translocation of pro-apoptotic mitochondrial proteins, and the feedback from effector to initiator caspases [60]. The up-regulation of several apoptosis genes in liver of riboflavin-deficient embryos likely contributes to their sudden death between e13 and e15.

From our transcriptional analyses of riboflavin deficiency in chick embryos, we discovered over-expression of porimin (*PORIMIN*), a novel gene encoding an oncosis-inducing receptor [61]. Oncotic cell death results from cell swelling and increased permeability of the cell membrane, which could have dire consequences for hepatocytes and endothelial cells lining blood capillaries alike. PORIMIN-induced disruption of blood capillaries in the riboflavin-deficient chick embryos could lead to the massive hemorrhage of skin and visceral organs observed at e15. Our qRT-PCR analysis of *PORIMIN* clearly shows over-expression of this novel gene in the liver of riboflavin-deficient embryos at e11, e13 and e15. There is a general consensus that oncosis is caused by failure of ion pumps due to lack of cellular ATP, which could be the case in riboflavin-deficient embryos that abruptly die from hypoglycemia [16].

## Conclusions

Our analysis of the liver in *rd/rd* embryos, with or without riboflavin rescue, across four embryonic ages (e9-e15) provides the first global view of the transcriptional responses to riboflavin deficiency and illustrates the importance of riboflavin availability for vital biological processes (i.e., β-oxidation of lipid, euglycemia, hemostasis and feathering). Riboflavin deficiency causes sudden death of chick embryos between e13 and e15 of development. However, injection of riboflavin before onset of the incubation of fertile eggs rescues *rd/rd* embryos. The most obvious effects of riboflavin deficiency observed in the Rf- embryos were their smaller size, excessive lipid accumulation in liver, massive cutaneous and visceral hemorrhage, impaired feathering and sudden death. Transcriptional profiling of the liver with genome-wide oligo microarrays has revealed hundreds of genes that are differentially expressed between Rf + and Rf- embryos at e13 and e15. Major biological pathways affected by riboflavin deficiency include fatty acid metabolism, blood coagulation, ubiquination/proteasome activation, and programed cell death. These pathways are populated by genes that are either up-regulated in the Rf + embryo or up-regulated in the Rf- embryo. One important observation that still warrants exploration is the correlation between reduced expression of *FKER* in liver of the Rf- embryo and their impaired cutaneous feathering. Although Rf- embryos are able to transport and store lipids from the yolk sac, they are unable to utilize hepatic lipids due to inhibition of riboflavin-dependent enzymes required for β-oxidation of fatty acid stores. It is obvious from the variety of biological processes affected by the *lack* of riboflavin that death of Rf- embryos between e13 and e15 is due to energy depletion and the complete shutdown of the various critical biological pathways. The generalized disruption of blood coagulation in riboflavin-deficient embryos and the demonstrated restoration of hemostasis gene expression by riboflavin replacement suggest that many genes in the blood clotting cascade are either directly or indirectly dependent upon riboflavin. However, the mechanisms by which the flavoproteome exerts these broad effects on metabolism, hemostasis and normal embryonic development of the chicken remain to be elucidated.

Nonetheless, our time-course transcriptional analyses of liver in riboflavin-deficient and riboflavin-rescued (*rd/rd*) embryos derived from the same genotype (*rd/rd*) of SCWL (egg-type) chickens clearly demonstrates the central importance of a single required nutrient (riboflavin or Vitamin B2) for normal growth and development of the chicken embryo. Essentially during the last half of rapid embryonic growth—a critical period exclusively fueled by oxidation of yolk lipids transported into liver. The metabolic collapse and sudden death of the riboflavin-deficient *rd/rd* embryos impacts a range of vital biological processes directly or indirectly responsive to the riboflavin-deficient embryo’s inability to oxidize yolk lipids, amassed in the liver. The present transcriptional study provides an excellent example of the maternal effect, where the mother’s genotype (*rd/rd*) determines the survival of embryos, while the embryo’s genotype (*rd/rd*) has little effect on growth and development after hatching. Thus, there is an absolute requirement for a functional riboflavin binding protein (RBP) in the hen for transport of essential riboflavin into the fertilized egg prior to oviposition, incubation and embryonation.

## Additional files

Additional file 1: Experimental design of microarray hybridizations. A Microsoft Excel file containing a single work sheet “Array Hybridization Design” which describes the hybridization scheme for 32 Arizona 20.7 K chicken oligo arrays used for the riboflavin deficient chick embryo study. (XLSX 15 kb)

Additional file 2: Primers used for qRT-PCR analysis. A Microsoft Excel file containing a single work sheet “qRT-PCR Primer Information”. This table provides the chicken gene symbol, Roslin Institute *Gallus gallus* (RIGG) oligo ID number, 5′-3′ sequence for forward and reverse primers, and amplicon size (bp) for each gene used for qRT-PCR analysis. (XLSX 16 kb)

Additional file 3: Differentially-expressed (Adjusted *P* ≤ 0.05; FDR ≤ 0.05) genes identified in liver of e13 and e15 embryos. A Microsoft Excel file containing two worksheets. Work sheets “Riboflavin e13_396 DE genes” and “Riboflavin e15_1467 DE genes” list information about differentially expressed genes determined by microarray analysis. Each list provides the Roslin Institute *Gallus gallus* (RIGG) oligo ID, gene symbol, gene description, log2 fold change (Rf+/Rf-), average expression (Ave/Expr), t-statistic, *P*-value, *P*-value adjusted for multiple testing (adj.*P* ≤ 0.05), B-statistic from Limma software, and the Ref-Seq peptide ID for each gene (oligo). (XLSX 328 kb)

Additional file 4: Lists of “Analysis Ready” DE genes (AR-DE genes) assigned by IPA to “Canonical Pathways”. A Microsoft Excel file containing a five worksheets of the top “Canonical Pathways” identified by IPA in riboflavin-rescued and riboflavin deficient chick embryos on embryonic day 15 (e15). Five major canonical pathways identified by IPA among DE genes on embryonic day 15 (e15) were: “EIF2 Signaling, Acute-phase Signaling, LXR-RXR Activation, FXR-RXR Activation, Intrinsic Prothrombin Activation”, and “Blood Clotting” in the “Biological Function Category” of IPA. Each worksheet provides the gene symbol, Entrez gene name, gene expression as log2 ratio (Rf+/Rf-), and Ref-Seq protein ID or Entrez gene ID. (XLSX 27 kb)

Additional file 5: IPA “Molecular and Cellular Functions” of AR-DE genes in riboflavin-rescued and riboflavin deficient chick embryos on embryonic day 15 (e15). A Microsoft Excel file containing nine worksheets of over-populated “Molecular and Cellular Functions” identified by IPA: “Fatty Acid Metabolism, Oxidation of Lipid, Carbohydrate (CHO) Metabolism, Catabolism of Protein, Metabolism of Amino Acids (AA), Protein Ubiquitination, Metabolism of Vitamins” and 29 AR-DE genes found in the chicken “*Flavoproteome*”. Each worksheet provides protein ID, gene symbol and gene expression as log2 ratio (Rf+/Rf-). (XLSX 35 kb)

Additional file 6: Pearson’s correlation analysis of gene expression levels of 21 DE “candidate” genes determined by 20.7 K microarray analysis and by qRT-PCR analysis. A Microsoft Excel file containing normalized expression levels (log2 ratio of Rf+/Rf-) of 21 DE candidate genes averaged across e13 and e15 as determined by both microarray analysis and qRT-PCR analysis. The Pearson’s correlation coefficient (*r* = 0.834; 19 degrees of freedom) indicates a highly significant (*P* ≤ 0.01) correlation between gene expression levels obtained from both microarray and qRT-PCR analyses. (XLSX 13 kb)

## Abbreviations

ANOVA: Analysis-of-variance
aRNA: Amplified RNA
BSA: Bovine serum albumen
Ct: Average cycle time
DE: Differentially expressed
DIG: Digoxigenin
DTT: Dithiothreitol
e9: Embryonic day 9
FAD: Flavin-adenine dinucleotide
FDR: False discovery rate
FMN: Flavin mononucleotide (or riboflavin-5′-phosphate)
GEO: Gene expression omnibus
GLM: General linear model
GPR: GenePix report file
IPA: Ingenuity Pathway Analysis
LSD: Least significant difference
MCAD: Medium-chain acyl-Coenzyme A dehydrogenase
MCADD: Medium-chain acyl-coenzyme A dehydrogenase disorder
MIAME: Minimum information about a microarray experiment
NCBI: National Center for Biotechnology Information
qRT-PCR: Quantitative reverse transcriptase polymerase chain reaction
QSOX: Quiescin sulfhydryl oxidase
RBP: Riboflavin-binding protein
*rd/rd*: Riboflavin binding protein-deficient hens
Rf: Riboflavin
Rf-: Riboflavin-deficient
Rf+: Riboflavin-rescued
RFVT1: Riboflavin transporter 1
RH: Relative humidity
RIGG: Roslin Institute *Gallus gallus*
RIN: RNA integrity number
SAS: Statistical Analysis System
SCWL: Single-comb White Leghorn
SDS: Sodium dodecyl sulfate
SSC: Saline sodium citrate

## References

[1] Romanoff AL (1967). The avian embryo.

[2] Tranter HS, Board RG (1982). The antimicrobial defense of avian eggs: biological perspective and chemical basis. J Appl Biochem.

[3] Du J, Hincke MT, Rose-Martel M, Hennequet-Antier C, Brionne A, Cogburn LA, Nys Y, Gautron J (2015). Identifying specific proteins involved in eggshell membrane formation using gene expression analysis and bioinformatics. BMC Genomics.

[4] Jonchere V, Rehault-Godbert S, Hennequet-Antier C, Cabau C, Sibut V, Cogburn LA, Nys Y, Gautron J (2010). Gene expression profiling to identify eggshell proteins involved in physical defense of the chicken egg. BMC Genomics.

[5] Moran ET (2007). Nutrition of the developing embryo and hatchling. Poult Sci.

[6] Speake BK, Murray AMB, Noble RC (1998). Transport and transformations of yolk lipids during development of the avian embryo. Prog Lipid Res.

[7] Maw AJG (1954). Inherited riboflavin deficiency in chicken eggs. Poult Sci.

[8] Buss EG, Carter TC, Freeman BM (1969). Genetic interference in the egg transfer, utilization, and requirement of riboflavin by the avian embryo. The fertility and hatchability of the avian egg.

[9] White HB, Merrill AH (1988). Riboflavin-binding proteins. Ann Rev Nutr.

[10] MacLachlan I, Nimpf J, White HB, Schneider WJ (1993). Riboflavinuria in the rd chicken. 5′-splice site mutation in the gene for riboflavin-binding protein. J Biol Chem.

[11] Winter WP, Buss EG, Clagett CO, Boucher RV (1967). The nature of the biochemical lesion in avian renal riboflavinuria. II. The inherited change of a riboflavin-binding protein from blood and eggs. Comp Biochem Physiol.

[12] Hoober KL, Joneja B, White HB, Thorpe C (1996). A sulfhydryl oxidase from chicken egg white. J Biol Chem.

[13] White HB, Nuwaysir EF, Komara SP, Anderson DA, Chang SJ, Sherwood TA, Alphin RL, Saylor WW (1992). Effect of riboflavin-binding protein deficiency on riboflavin metabolism in the laying hen. Arch Biochem Biophys.

[14] White HB (1996). Sudden death of chicken embryos with hereditary riboflavin deficiency. J Nutr.

[15] Decuypere E, Nouwen EJ, Kuhn ER, Geers R, Michels H (1979). Iodohormones in the serum of chick embryos and post-hatching chickens as influenced by incubation temperature. Relationship with the hatching process and thermogenesis. Ann Biol Anim Biochim Biophys.

[16] Abrams VAM, Han CC, White HB (1995). Riboflavin-deficient chicken embryos: hypoglycemia without dicarboxylic aciduria. Comp Biochem Physiol B Biochem Mol Biol.

[17] Pugh E, Sidbury JB (1971). Fatty acid oxidation in embryonic chick tissues. Biochim Biophys Acta.

[18] Bennett MJ, Allison F, Pollitt RJ, Variend S, Tanaka K, Coates PM (1990). Fatty acid oxidation defects as causes of unexpected death in infancy. Fatty acid oxidation: clinical, biochemical and molecular aspects.

[19] Barile M, Giancaspero TA, Leone P, Galluccio M, Indiveri C (2016). Riboflavin transport and metabolism in humans. J Inherit Metab Dis.

[20] Bourin M, Gautron J, Berges M, Hennequet-Antier C, Cabau C, Nys Y, Réhault-Godbert S (2012). Transcriptomic profiling of proteases and antiproteases in the liver of sexually mature hens in relation to vitellogenesis. BMC Genomics.

[21] Wang X, Newkirk RF, Carre W, Ghose P, Igobudia B, Townsel JG, Cogburn LA (2009). Regulation of ANKRD9 expression by lipid metabolic perturbations. BMB Rep.

[22] ARK-Genomics Centre for Comparative & Functional Genomics. http://www.ark-genomics.org/. Accessed 17 Sept 2017.

[23] Trakooljul N, Konieczka JH, Hoying A, Antin PB, Porter TE, Cogburn LA: NCBI GEO Platform GPL6049. Arizona *Gallus gallus* 20.7K Oligo Array v1.0. https://www.ncbi.nlm.nih.gov/geo/query/acc.cgi?acc=GPL6049. Accessed 26 Oct 2007.

[24] Casel P, Moreews F, Lagarrigue S, Klopp C (2009). sigReannot: an oligo-set re-annotation pipeline based on similarities with the Ensembl transcripts and Unigene clusters. BMC Proc.

[25] Cogburn Laboratory. http://cogburn.dbi.udel.edu/index.html. Accessed 26 Oct 2017.

[26] Smyth GK, Gentleman R, Carey V, Dudoit S, Irizarry R, Huber W (2005). Limma: linear models for microarray data. Bioinformatics and computational biology solutions using R and Bioconductor.

[27] Benjamini Y, Hochberg Y (1995). Controlling the false discovery rate: a practical and powerful approach to multiple testing. J Royal Stat Soc (Series B).

[28] Rosa GJM, Steibel JP, Tempelman RJ (2005). Reassessing design and analysis of two-colour microarray experiments using mixed effects models. Comp Funct Genom.

[29] Tempelman RJ (2005). Assessing statistical precision, power, and robustness of alternative experimental designs for two color microarray platforms based on mixed effects models. Vet Immunol Immunopath.

[30] Resnyk CW, Carré W, Wang X, Porter TE, Simon J, Le Bihan-Duval E, Duclos MJ, Aggrey SE, Cogburn LA (2013). Transcriptional analysis of abdominal fat in genetically fat and lean chickens reveals adipokines, lipogenic genes and a link between hemostasis and leanness. BMC Genomics.

[31] Resnyk CW, Carré W, Wang X, Porter TE, Simon J, Le Bihan-Duval E, Duclos MJ, Aggrey SE, Cogburn LA (2017). Transcriptional analysis of abdominal fat in chickens divergently selected on bodyweight at two ages reveals novel mechanisms controlling adiposity: validating visceral adipose tissue as a dynamic endocrine and metabolic organ. BMC Genomics.

[32] Ingenuity Pathway Analysis. https://www.qiagenbioinformatics.com/products/ingenuity-pathway-analysis/. Accessed 07 Nov 2017.

[33] Biogazelle qbase+ software. https://www.qbaseplus.com/. Accessed 08 Mar 2017.

[34] Hellemans J, Mortier G, De Paepe A, Speleman F, Vandesompele J (2007). qBase relative quantification framework and software for management and automated analysis of real-time quantitative PCR data. Genome Biol.

[35] Lee CM, White HBI (1996). Riboflavin-binding protein induces early death of chicken embryos. J Nutr.

[36] White HB, Ferguson MWJ, Deeming DC (1991). Maternal diet, maternal proteins, and egg quality. Egg incubation: its effects on embryonic development in birds and reptiles.

[37] Noble RC, Cocchi M (1990). Lipid metabolism and the neonatal chicken. Prog Lipid Res.

[38] Wang M, Liu YE, Goldberg ID, Shi YE (2003). Induction of mammary gland differentiation in transgenic mice by the fatty acid-binding protein MRG. J Biol Chem.

[39] Bauer R, Plieschnig JA, Finkes T, Riegler B, Hermann M, Schneider WJ (2013). The developing chicken yolk sac acquires nutrient transport competence by an orchestrated differentiation process of its endodermal epithelial cells. J Biol Chem.

[40] Demeure O, Duby C, Desert C, Assaf S, Hazard D, Guillou H, Lagarrigue S (2009). Liver X receptor {alpha} regulates fatty acid synthase expression in chicken. Poult Sci.

[41] Gomez-Ospina N, Potter CJ, Xiao R, Manickam K, Kim MS, Kim KH, Shneider BL, Picarsic JL, Jacobson TA, Zhang J, He W, Liu P, Knisely AS, Finegold MJ, Muzny DM, Boerwinkle E, Lupski JR, Plon SE, Gibbs RA, Eng CM, Yang Y, Washington GC, Porteus MH, Berquist WE, Kambham N, Singh RJ, Xia F, Enns GM, Moore DD (2016). Mutations in the nuclear bile acid receptor FXR cause progressive familial intrahepatic cholestasis. Nat Commun.

[42] Fu Z, Runquist JA, Forouhar F, Hussain M, Hunt JF, Miziorko HM, Kim JJ (2006). Crystal structure of human 3-Hydroxy-3-methylglutaryl-CoA lyase: insights into catalysis and the molecular basis for hydroxymethylglutaric aciduria. J Biol Chem.

[43] Levi M, van der Poll T (2017). Coagulation and sepsis. Thrombosis Res.

[44] Rao LVM, Pendurthi UR (2012). Regulation of tissue factor coagulant activity on cell surfaces. J Thromb Haemost.

[45] O'Brien JM, Williams A, Yauk CL, Crump D, Kennedy SW (2013). In vitro microarray analysis identifies genes in acute-phase response pathways that are down-regulated in the liver of chicken embryos exposed *in ovo* to PFUdA. Toxicol in Vitro.

[46] Lienhart W-D, Gudipati V, Macheroux P (2013). The human flavoproteome. Arch Biochem Biophys.

[47] Hawes RO, Buss EG (1965). The use of the riboflavinless gene (*rd*) in determining the cause of clubbed down. Poultry Sci.

[48] Whitehead CC, McCormack HA, Hocking PM (1993). Defective down syndrome in chicks is not caused by riboflavin deficiency in breeders. Brit. Poult Sci.

[49] Cogburn LA, Wang X, Carré W, Rejto L, Aggrey SE, Duclos MJ, Simon J, Porter TE (2004). Functional genomics in chickens: development of integrated-systems microarrays for transcriptional profiling and discovery of regulatory pathways. Comp Funct Genom.

[50] Carré W, Wang X, Porter TE, Nys Y, Tang J-S, Bernberg E, Morgan R, Burnside J, Aggrey SE, Simon J, Cogburn LA (2006). Chicken genomics resource: sequencing and annotation of 35,407 chicken ESTs from single and multiple tissue cDNA libraries and CAP3 assembly of a chicken gene index. Physiol Genomics.

[51] Greenwold M, Sawyer R (2010). Genomic organization and molecular phylogenies of the beta (beta) keratin multigene family in the chicken (Gallus gallus) and zebra finch (Taeniopygia guttata): implications for feather evolution. BMC Evol Biol.

[52] Gillespie M, Crowley T, Haring V, Wilson S, Harper J, Payne J, Green D, Monaghan P, Donald J, Nicholas K, Moore R (2013). Transcriptome analysis of pigeon milk production - role of cornification and triglyceride synthesis genes. BMC Genomics.

[53] Pankov RG, Uschewa AA, Tasheva BT, Markov GG (1987). Vertebrate liver cytokeratins: a comparative study. Biochem Cell Biol.

[54] Prum R, Brush A (2002). The evolutionary origin and diversification of feathers. Q Rev Biol.

[55] Lin CM, Jiang TX, Widelitz RB, Chuong CM (2006). Molecular signaling in feather morphogenesis. Curr Opin Cell Biol.

[56] Chuong CM, Homberger DG (2003). Development and evolution of the amniote integument: current landscape and future horizon. J Exp Zool B Mol Dev Evol.

[57] Toni M, Dalla Valle L, Alibardi L (2007). The epidermis of scales in gecko lizards contains multiple forms of β-keratins including basic glycine-proline-serine-rich proteins. J Proteome Res.

[58] Bao W, Greenwold MJ, Sawyer RH (2016). Expressed miRNAs target feather related mRNAs involved in cell signaling, cell adhesion and structure during chicken epidermal development. Gene.

[59] Taborsky G, Mok CC (1967). Phosvitin: homogeneity and molecular weight. J Biol Chem.

[60] Albeck JG, Burke JM, Aldridge BB, Zhang M, Lauffenburger DA, Sorger PK (2008). Quantitative analysis of pathways controlling extrinsic apoptosis in single cells. Mol Cell.

[61] Ma F, Zhang C, Prasad KVS, Freeman GL, Schlossman SF (2001). Molecular cloning of Porimin, a novel cell surface receptor mediating oncotic cell death. Proc Natl Acad Sci U S A.

